# Comparative Analysis of Functional Metagenomic Annotation and the Mappability of Short Reads

**DOI:** 10.1371/journal.pone.0105776

**Published:** 2014-08-22

**Authors:** Rogan Carr, Elhanan Borenstein

**Affiliations:** 1 Department of Genome Sciences, University of Washington, Seattle, WA, United States of America; 2 Department of Computer Science and Engineering, University of Washington, Seattle, WA, United States of America; 3 Santa Fe Institute, Santa Fe, NM, United States of America; University of North Carolina at Charlotte, United States of America

## Abstract

To assess the functional capacities of microbial communities, including those inhabiting the human body, shotgun metagenomic reads are often aligned to a database of known genes. Such homology-based annotation practices critically rely on the assumption that short reads can map to orthologous genes of similar function. This assumption, however, and the various factors that impact short read annotation, have not been systematically evaluated. To address this challenge, we generated an extremely large database of simulated reads (totaling 15.9 Gb), spanning over 500,000 microbial genes and 170 curated genomes and including, for many genomes, *every* possible read of a given length. We annotated each read using common metagenomic protocols, fully characterizing the effect of read length, sequencing error, phylogeny, database coverage, and mapping parameters. We additionally rigorously quantified gene-, genome-, and protocol-specific annotation biases. Overall, our findings provide a first comprehensive evaluation of the capabilities and limitations of functional metagenomic annotation, providing crucial goal-specific best-practice guidelines to inform future metagenomic research.

## Introduction

Metagenomics, the study of uncultured microorganisms through the analysis of genomic material obtained directly from environmental samples, has been used to study the structure, function, and dynamics of microbial communities. These studies allowed us to gain a deeper understanding of the tremendous diversity of microbes in various environments [1]–[3], the complex structure of the communities they form [4], and the crucial impact they have on the surrounding environment [5]. Amongst the numerous communities explored by metagenomics-based methods, studies of the human microbiome have been especially exciting, allowing us to characterize the previously unmapped composition of this complex ecosystem [4], [6]–[8] and to link compositional shifts in the microbiome to multiple diseases [6], [9]–[11].

While earlier studies focused mainly on the composition of species in the community, nowadays, many metagenomic studies take a gene-centric approach and further aim to characterize the overall functional profile of the community through shotgun metagenomics. In such studies, shotgun sequencing reads are mapped to a database of orthologous gene groups (e.g., KEGG [12], COG [13], EggNOG [14], M5NR [15], Metacyc [16]) to identify matches to genes or proteins with known and annotated functions. Notably, it is the biological function of the identified homologous gene rather than the taxonomic identity that is sought in such an analysis. Accordingly, mapping is most often done with a translated BLAST search to identify high-scoring (though potentially evolutionarily distant) alignments indicative of orthology [17]. By tracking the number of reads that map to each gene family or orthology group, the functional profile of the metagenome can be obtained, providing insight into the functional capacity of the community as a whole [4], [7], [11], [18]–[20]. This annotation process and the estimation of the number of reads that map to each function is a crucial step in comparative metagenomic studies and in the discovery of functional shifts associated with disease, as it is the foundation of all such analyses.

A critical assumption behind this annotation scheme and the key to accurately recovering the functional profile of the metagenome is that short sequencing reads contain enough information to unambiguously map to the correct function. Put differently, these annotation protocols assume that each read will be mapped to either its gene of origin (if, for example, a genome closely related to the one from which the read originated were to be present in the database) or to an orthologous gene from a potentially distant genome annotated with the same function. These protocols further assume that reads originating from intergenic regions or from orthology groups with unknown function (e.g., functions not represented in our database) will not be erroneously mapped to any of the functionally annotated genes in our database. These assumptions are essential both for estimating the relative abundance of each function (or gene) in the community and for determining the presence or absence of gene families or other functional modules of interest. To date, however, no comprehensive and systematic analysis has evaluated these assumptions on a large scale, nor have protocols for this annotation procedure been standardized. The lack of such a systematic analysis makes the results presented in many metagenomic studies hard to assess and interpret and challenges any attempt to compare functional analysis across different studies.

Notably, the assumptions above and the annotation of short reads are still important even when some preprocessing (e.g., clustering [21], [22] or assembly [4], [6], [23], [24]) or post processing (e.g., pathway-level analysis or network reconstruction [20], [25]) are involved. Specifically, when short reads are clustered or assembled into contigs and whole genes are called and annotated, direct short-read annotation can still be used to annotate the potentially sizeable number of unassembled reads (e.g. ∼50% of reads from human microbiome samples [6], [26]). Moreover, even when reads are assembled into contigs, direct functional annotation is often still performed independently for *all* short reads to obtain an unbiased functional abundance profile for the entire sample [4]. Similarly, when estimating pathway coverage and abundance, even though the integration of annotation data from multiple reads, gap filling methods, and subnetwork scoring schemes can improve the accuracy of the obtained functional profile, the accuracy of the annotation of individual reads will clearly impact any downstream analysis. More generally, characterizing the information content of short reads and their mappability to gene families and functions goes beyond addressing questions concerning the applicability of short read annotation practices and may help elucidate profound and fundamental properties of microbial genomes.

Here, we address this challenge and perform a large-scale and systematic evaluation of homology-based functional annotation of short reads. We aim to uncover the impact of various technological and biological factors affecting the resulting annotation profiles, to compare common annotation protocols, and, more generally, to investigate the intrinsic mappability of such short reads. We focus primarily on annotation protocols that use translated BLAST. Direct translated BLAST searches of short reads for functional homology is a common strategy in microbiome research [4], [7], [11], [17]–[20], and was one of the key methods used by the Human Microbiome Project (HMP) for sample annotation [4], [20]. Tools promoted by this project (e.g., [20]) further relied on such BLAST alignments as input and are becoming the *de facto* standard in human microbiome research. To date, however, there has been no in-depth analysis of the systematic biases inherent to these homology-based annotation protocols and their many variations found in the literature. Our study, accordingly, seeks to provide a benchmark, demonstrating what can be expected of such annotation strategies, what their strengths and weaknesses are, and what biases can be expected in the functional annotation profiles obtained by such protocols.

To this end, we have constructed a large-scale database containing more than 143 million simulated shotgun sequencing reads spanning more than 500,000 microbial genes and 170 curated and annotated genomes. Each read was annotated using common metagenomic protocols, and the obtained annotations were carefully analyzed at various scales, from the single-read level to the entire dataset level, to investigate the different factors that affect the accuracy of the obtained annotations. We took advantage of the fact that BLAST-based annotation maps each read to the database independently to analyze the annotation obtained for each read in the context of the genome from which it originated, fixing in place any genomic and phylogenetic characteristics that might bias the annotation process and allowing us to accurately characterize the impact of such characteristics across the bacterial and archaeal tree of life. This approach additionally allowed us to test the importance of the phylogenetic coverage of the database (i.e., the relation of the strain in question to those present in the database) to the annotation process. We further rigorously examined the impact of sequencing error, read length, phylogeny, copy number, annotation protocol, and BLAST parameters. Finally, we examined the effect of different annotation protocols on the functional profile obtained for HMP metagenomic samples, and assessed the performance of an alternative alignment strategy. Overall, this extensive analysis provides a first comprehensive assessment of functional metagenomic annotation, quantifying gene orthology group-specific, genome-specific, and protocol-specific annotation biases and suggesting goal-dependent best-practice guidelines.

## Results

### Reference genomes, simulated short reads, and annotations

We collected curated reference genomes of 170 microbial species spanning 23 phyla and 89 genera across the bacterial and archaeal tree of life (Table S1). Curated annotations of these genomes were obtained from the KEGG database [12] to identify protein-coding genes and their associated KEGG Orthology (KO) annotations. The total number of genes and the number of KO-associated genes (‘KO genes’) vary significantly across genomes (Table S1). For example, the genome of *E. coli* O157:H7, a well-characterized strain, was annotated with 5477 protein-coding genes, of which 2999 were KO genes (55%), while the genome of *B. thetaiotaomicron*, a human commensal bacterium, had 4816 protein-coding genes, of which only 1365 were KO genes (28%). The genome of *S. pneumoniae* ATCC 700669, which we analyze in detail below, was also relatively well characterized, with 55% of its protein-coding genes mapped to KOs. The *phylogenetic coverage* of these genomes in the KEGG database also vary significantly, with some having as many as 51 genomes from the same species (*E. coli*) and as many as 79 in the same genus (*S. pneumoniae*), while other genomes were the only representative of their genus in the database.

**Figure 1:**
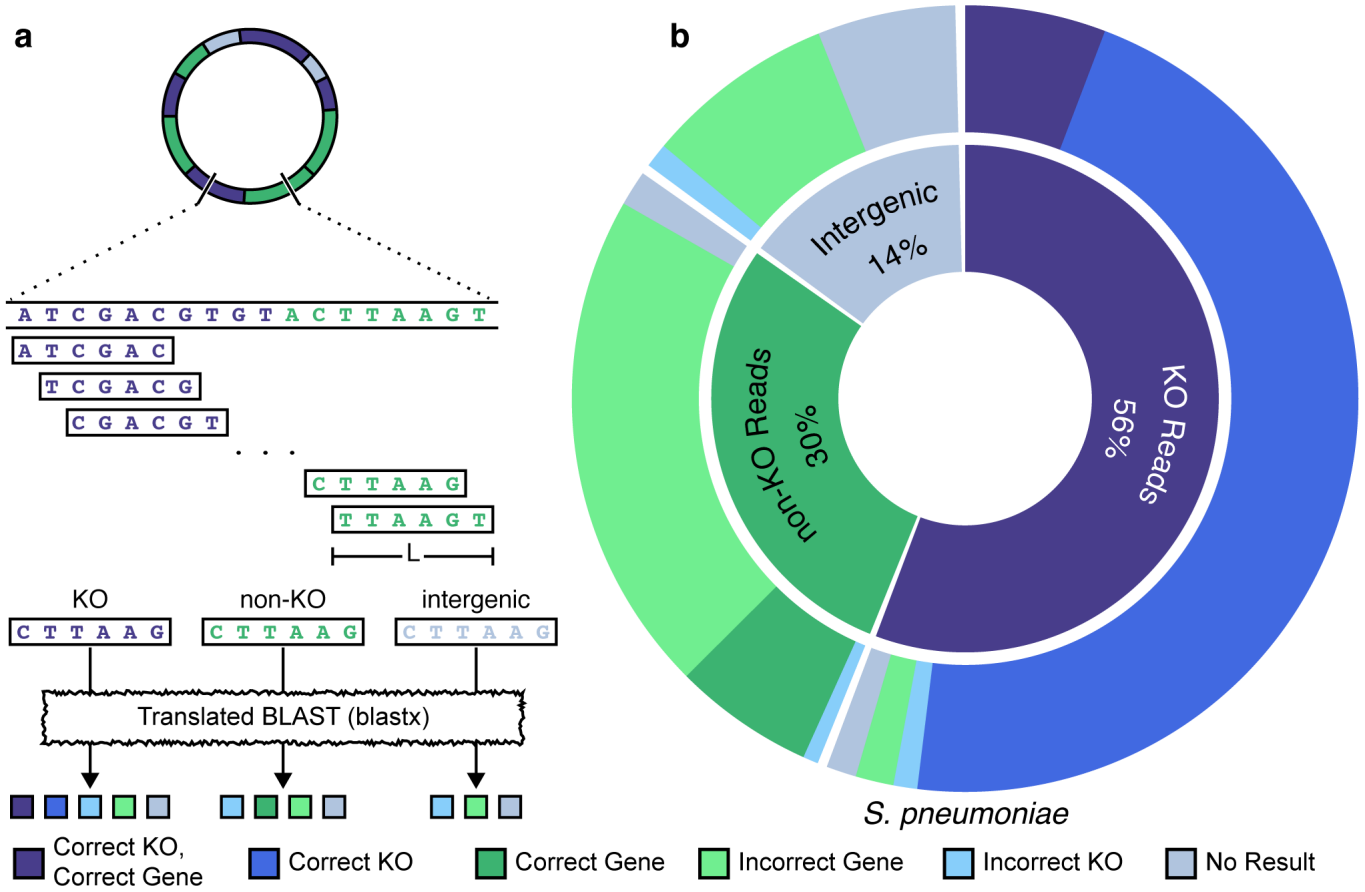
Overview of the analysis scheme. (**a**) Simulated sets of reads of length L are generated from curated and annotated reference genomes using a sliding window approach. The origin of each read is recorded, and reads are labeled to note whether they originated from genes associated with a KO (purple), from genes not associated with a KO (green), or from an intergenic region (gray). Each read is then annotated through a translated BLAST search against the KEGG database and the obtained annotation is compared to the annotation of the genome region from which the read was derived, to evaluate whether the correct gene and/or correct KO were recovered. (**b**) Evaluating the annotation of the *S. pneumoniae* genome. The inner ring represents the proportion of the genome annotated with KO genes, non-KO genes, and intergenic regions. The outer ring illustrates how reads originating from each such category were annotated, illustrating the accuracy of the annotations obtained by a BLAST-based search.

From these genomes we generated an extremely large-scale database of simulated short sequencing reads, including 179 unique short read datasets totaling more than 15.9 Gb (Table S2). Specifically, we used a sliding window approach to generate short reads of a given length from each of the genomes above. For some genomes, *every* possible 101-bp read was generated (Figure 1a). As described below, additional datasets were also generated to examine the impact of read length and sequencing error on the annotation process (Table S2). The origin of each read was recorded to note whether it originated from a KO gene, a non-KO gene, or an intergenic region. These reads were then mapped to the KEGG database using the blastx protocol employed by the Human Microbiome Project (HMP) [4], [20] and annotated according to the identified matches. To evaluate the accuracy of the annotation process, obtained annotations were compared to the annotation of the region in the curated genome from which each read originated (Figure 1a). Complete details of the methods used are provided in *Methods*.

### A best-case scenario for functionally annotating short reads

We first analyze an ideal case for annotating short shotgun metagenomic sequencing reads, wherein reads originated from strains that have been sequenced, are well-annotated, and are present in the reference database. We used the set of *every* possible *S. pneumoniae* read, described above, and annotated each read using a typical BLAST-based protocol (see below and *Methods*). Overall, we found that the obtained annotation accurately described the *S. pneumoniae* strain's known functions with little error (Figure 1b). Specifically, KO annotations had 96.9% precision, suggesting that only a few reads from non-KO genes or from intergenic regions were incorrectly assigned a KO and that only a few reads from KO genes were assigned the wrong KO. Moreover, the annotation of reads originating from a KO gene had a 94.7% recall, indicating that, generally, reads originating from such genes were correctly identified. Importantly however, in the majority of cases, while the correct KO was recovered, the annotation process could not accurately identify the specific gene of origin, as reads often matched equally well to homologous genes from multiple other strains (Figure 1b). The prevalence of such tied matches is at least partly an outcome of using a translated BLAST search which identifies matches at the peptide level, allowing divergence in the nucleotide sequence. This lack of specificity is a clear benefit of the translated BLAST approach in functionally annotating short reads, but comes at the cost of inaccurate taxonomic classification, for which marker gene approaches are better suited [27]–[29].

### The impact of sequencing errors and read length

Importantly, one of the key goals of this study is to characterize the information contained in short DNA fragments in terms of the function of the gene from which they originated. We therefore focused on the potential mappability of short reads and largely ignored artifacts introduced by specific sequencing technologies. Yet, to confirm that such technology-derived factors do not dramatically affect our findings, we examined the impact of typical sequencing errors on the annotation process. To this end, experimentally derived Illumina error profiles were added to the set of 101-bp *S. pneumoniae* reads with error rates of 0.15%, 1.5%, and 3% to generate 3 new datasets (*Methods*). Reads were then annotated and analyzed as before. Overall, we found a minimal change in the accuracy of the obtained annotations, with comparable recall and precision levels to those observed without sequencing errors, regardless of the error rate or phylogenetic coverage (Table S3).

Thus far, our analysis has been restricted to 101-bp reads – a common length for Illumina sequencing reads and the length generated by the HMP [26]. However, various sequencing technologies (e.g. Roche 454 Genome Sequencer, Pacific Biosciences, as well as newer Illumina protocols) can now generate longer reads, and in general, it is likely that available high-throughput reads will continue to increase in length as sequencing technologies mature. To examine the effect of read length on the performance of a typical translated BLAST-based annotation protocol, we generated 5 additional simulated datasets from the *S. pneumoniae* genome with read lengths of 75, 150, 200, 300, and 400 bp (Table S2). Annotating and analyzing these datasets using the same protocols as before, we found, not surprisingly, that read length significantly influences the annotation process. Specifically, for reads originating from within KO genes, the recall increased with read length, while the precision decreased (Table S3). Including all reads overlapping KO genes, recall begins to decrease with read length for reads longer than 150 bp (Figure S1b). Furthermore, the choice of annotation protocol (see below) significantly affects annotation performance for longer reads (Table S3). Current protocols were developed mostly for 101-bp or shorter reads and consequently commonly used alignment cutoff values (e.g., E-value<1) may be too permissive for longer reads, leading to spurious annotations and reduced precision (Text S1 and Figure S1). Examining the E-value distributions obtained for longer reads demonstrated that as expected, for a fixed E-value cutoff, precision decreases with read length (Figure S1a, Figure S2). Moreover, length-dependent cutoff values can be determined to increase the annotation precision with a relatively small decrease in recall (Text S1).

### The impact of phylogenetic coverage of the reference database

**Figure 2:**
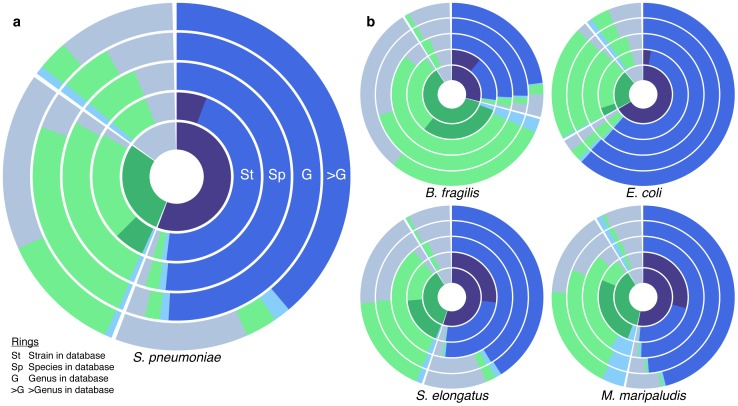
The impact of phylogenetic coverage of the database on the performance of a translated BLAST-based annotation. (**a**) The inner ring represents the proportion of the *S. pneumonia* genome annotated with KO genes, non-KO genes, and intergenic regions, as in Figure 1. Outer Rings illustrate the annotations for reads from each such category obtained with varying levels of phylogenetic coverage, ranging from having the strain from which the reads originated present in the database, to having only other genomes from the same species, genus, or higher taxonomic levels present. (**b**) The impact of phylogenetic database coverage on the annotation of *B. fragilis*, *E. coli*, *S. elongatus*, and *M. maripaludis* genomes.

Clearly, in many — if not most — cases, the actual strains from which the sampled microbial reads originate are not represented in the reference database. In fact, in many environments, the community may include strains from species, genera, or even higher taxonomic levels that are not at all covered by reference genomes [30]. It is therefore crucial to test the robustness of the annotation process in such scenarios and to assess how well short reads can be functionally mapped in the absence of closely related references. To this end, we re-annotated the set of short reads derived from *S. pneumonia* described above, but with the corresponding (i) strain, (ii) species, or (iii) genus removed from the database (*Methods*). We found that predicted annotations decreased in accuracy with phylogenetic coverage as expected (Figure 2a). However, as long as genomes from the genus *Streptococcus* were present in the database, the impact on the annotation process was relatively minimal. In contrast, when all genomes from the genus *Streptococcus* were removed from the database, the recall fell to 70.7%.

**Figure 3:**
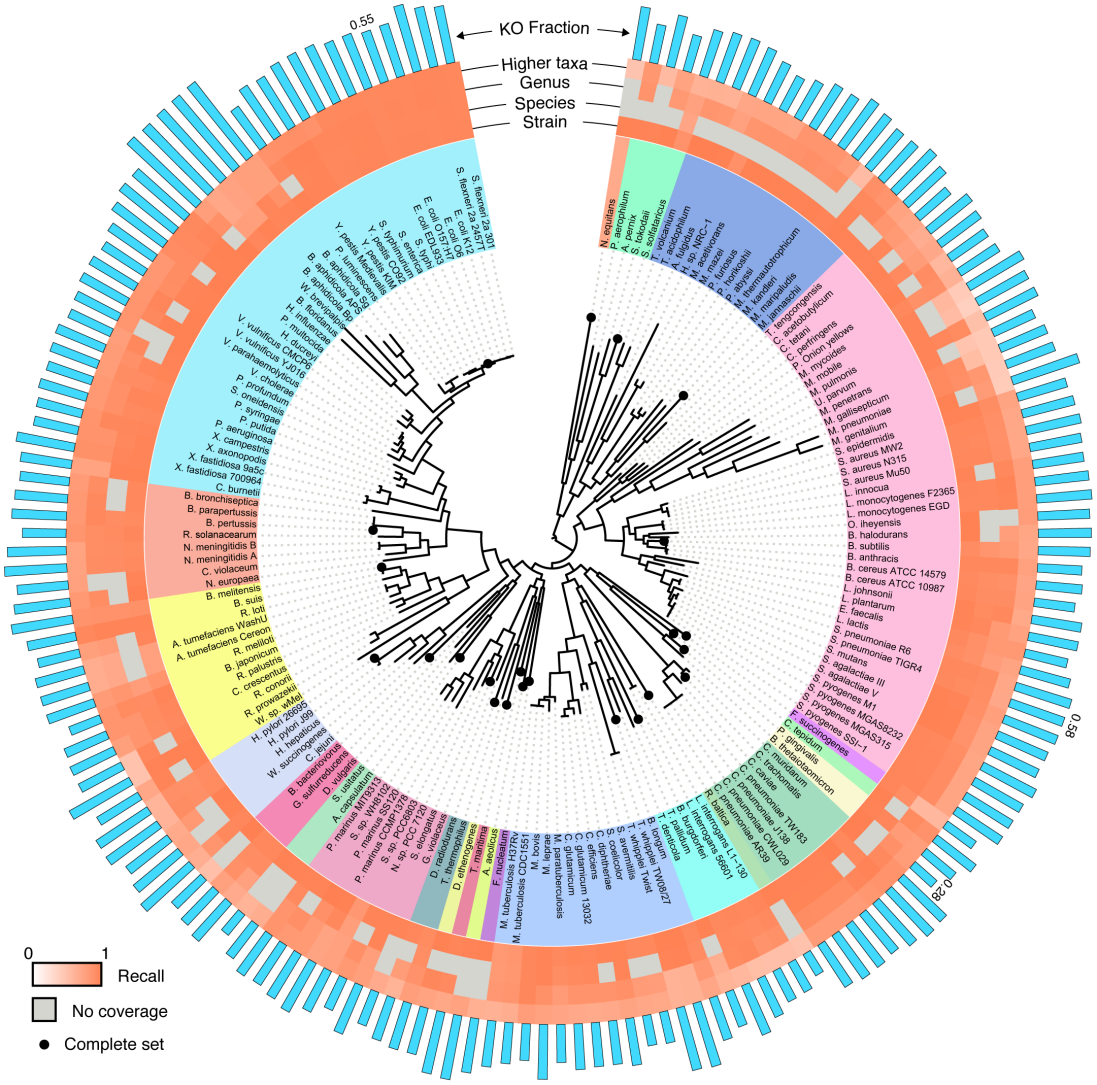
The performance of BLAST-based annotation of short reads across the bacterial and archaeal tree of life. The phylogenetic tree was obtained from Ref. [50]. Colored rings represent the recall for identifying reads originating from a KO gene using the *top gene* protocol. The 4 rings correspond to varying levels of database coverage. Specifically, the innermost ring illustrates the recall obtained when the strain from which the reads originated is included in the database, while the other 3 rings, respectively, correspond to cases where only genomes from the same species, genus, or more remote taxonomic relationships are present in the database. Entries where no data were available (for example, when the strain from which the reads originated was the only member of its species) are shaded gray. For one genome in each phylum, denoted by a black dot at the branch tip, every possible 101-bp read was generated for this analysis. For the remaining genomes, every 10^th^ possible read was used. Blue bars represent the fraction of the genome's peptide genes associated with a KO; for reference, the values are shown for *E. coli*, *B. thetaiotaomicron*, and *S. pneumoniae*.

Notably, *S. pneumoniae* is well covered by our database, which contains 18 genomes from this species and 79 genomes from the genus *Streptococcus*. To determine whether the results described above could be generalized to other bacterial and archaeal species, we performed the same analysis for 4 additional species: *Bacteroides fragilis*, *Escherichia coli* (both human commensal bacteria), *Synechococcus elongatus* (a freshwater cyanobacterium), and *Methanococcus maripaludis* (a marine archaeon). The impact of phylogenetic coverage for these 4 species is generally consistent with that seen in *S. pneumonia*, with overall high recall and precision for mapping reads from KO genes when the strain is present in the database and a decrease in performance with decreasing phylogenetic coverage (Figure 2b). However, the exact decrease and the coverage at which accuracy dropped significantly, vary from species to species. For example, the recall for annotating *S. elongatus* drops dramatically even after the removal of all the species genomes from the database, although other members of this genus are still present in the database. Additional results for the impact of phylogenetic coverage across a large array of microbial organisms are presented below (and in Figure 3).

### Annotation accuracy across the tree of life

To fully explore the effect of phylogeny on translated BLAST-based short read annotation of bacteria and archaea, we performed the same analysis for an additional 165 organisms across the bacterial and archaeal tree of life (Figure 3). For one organism from each phylum, every possible 101-bp read was simulated, and for the remaining organisms every 10^th^ possible read was simulated (i.e., using a sliding window with 10-bp jumps). We found that the precision for identifying reads from KO genes was generally consistent across the tree (average precision 0.95±0.03), but the recall appears to be clade specific (Figure 3). Furthermore, the effect of the phylogenetic coverage of the database on recall also varied significantly from clade to clade. Interestingly, while some of this variation could be attributed to the availability of reference genomes closely related to the clade of interest (e.g. *Escherichia*, *Shewanella*), some of the variation is clearly attributed to natural divergence within the clade. For example, as mentioned above, the annotation of reads from a *Synechococcus elongatus* genome is relatively poor when *elongatus* species are removed from the database even when other genomes from the same genus are present. This is possibly because the genus *Synechococcus* contains both freshwater and marine species, and *S. elongatus*, a freshwater species, has diverged significantly from the marine species included in this clade. Such clade-specific variations should be taken into account when annotating short reads from relatively less well studied clades.

### Comparison of annotation protocols

In most studies, the translated BLAST search is only the first step in the annotation process and one of a variety of protocols is then used to analyze the obtained set of alignments (i.e., those that satisfy a predefined threshold) and to determine the annotation that will be assigned to each read. For our analysis in the previous sections, we used the ‘*top gene’* protocol, assigning each read with the gene annotation of the top-scoring alignment(s). This is a common protocol that has been used in many previous studies (e.g., [7], [18], [31]–[33]). Other studies, however, apply different protocols, such as assigning the annotation of potentially sub optimal alignments to enrich for functional annotations [19], or averaging over multiple high-scoring hits (e.g., [4], [20]). In this section, we therefore compared the *top gene* protocol with three additional protocols: The ‘*top KO’* protocol aims to maximize the number of annotated reads, assigning the read with the annotation of the top-scoring alignment to a KO gene, even if better alignments have been found to other non-KO genes in the database. This is analogous to performing a search only against genes with known orthology groups (e.g., COG [13], EggNOG [14]). The *‘top 20 genes’* protocol seeks to correct for annotation errors and mismatches, using the annotation of the top 20 alignments weighted by their E-values. Finally, the *‘top 20 KOs’* protocol combines these two approaches, using the same top 20 alignments but considering only those that align to KO genes, weighted by E-value [20]. A detailed description of these protocols is provided in *Methods*.

**Figure 4:**
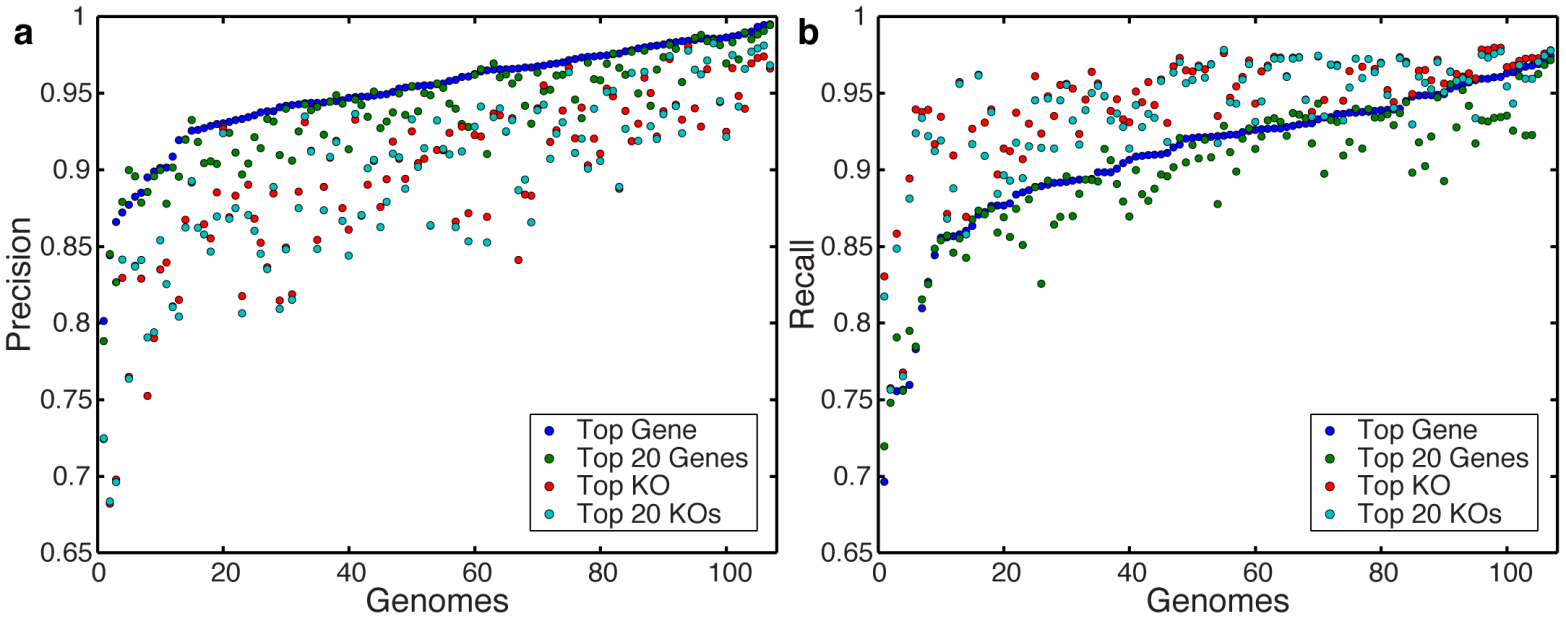
Comparison of annotation protocols for analyzing BLAST results. The (**a**) precision and (**b**) recall are illustrated for several protocols for identifying reads originating from KO genes when the strain from which the reads originated is absent from the database. Genomes are ordered by their precision and recall, respectively, using the *top gene* protocol.

To evaluate these annotation protocols, we examined the set of annotations obtained by each protocol for each of the 170 strain-derived datasets described above, calculating the precision and recall in each case (Table S3). We found marked differences between the performances of these four protocols, highlighting the impact of annotation methodology on downstream results. Specifically, when the strain from which the reads originated was removed from the BLAST database, the *top gene* protocol reached higher precision than any of the three other protocols in almost every dataset, with the *top 20 genes* protocol being a close second (Figure 4a). Yet, this high precision comes with slightly lower recall, and when higher recall is desired, the *top KO* or *top 20 KOs* are better choices (Figure 4b). These patterns hold regardless of the phylogenetic coverage of the database (Table S3). Generally, however, it appears that protocols that consider only alignments to KO genes (e.g., *top KO* and *top 20 KOs*) suffer a lack of precision in return for a slightly higher recall. This tradeoff between precision and recall can have a significant impact on downstream analysis and should be considered when designing a metagenomic analysis pipeline with a specific goal.

### Evaluating the annotation profile obtained for complete sets of reads

**Figure 5:**
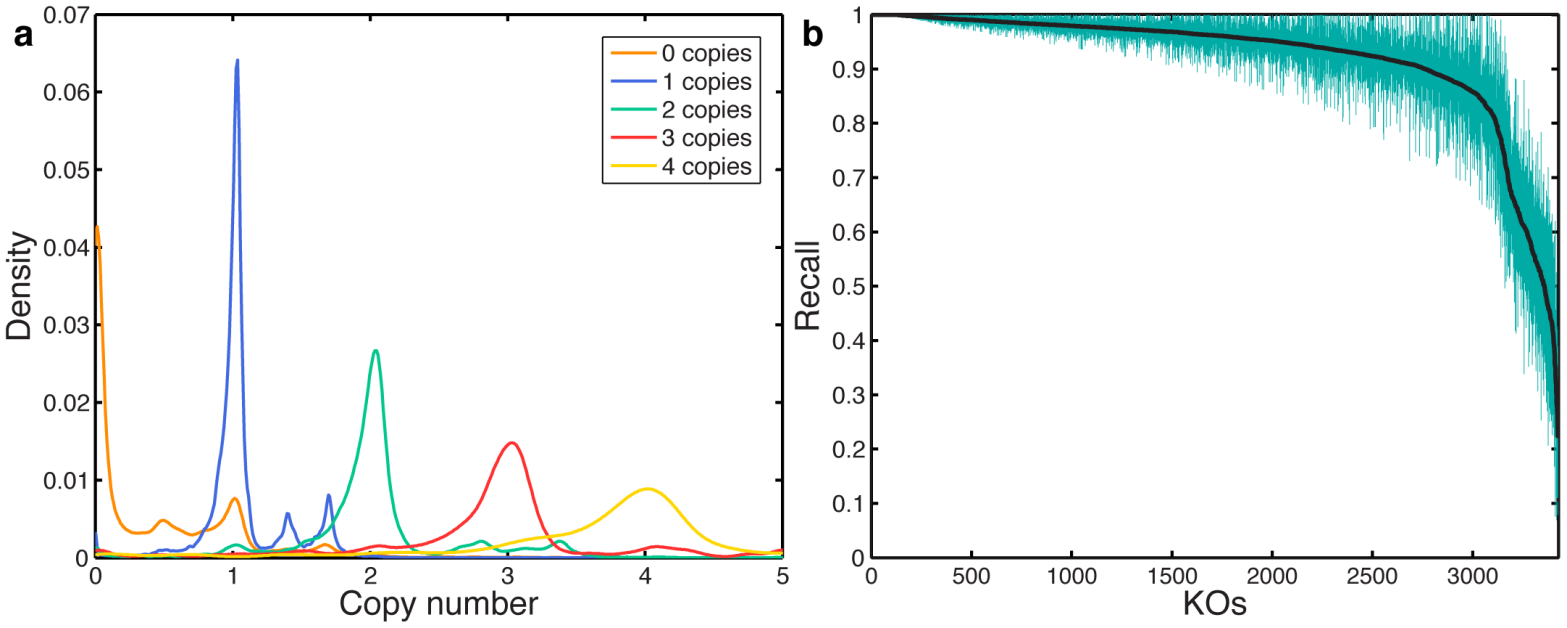
Performance of BLAST-based annotation in recovering the functional profile of a complete set of reads. (**a**) The probability function of predicting the copy number of a given KO in a given dataset across all simulated 101-bp datasets using the *top gene* protocol and when the strain from which the reads originated is absent from the database. Only KOs with copy numbers 1 to 4 are illustrated. The curve corresponding to copy number 0 represents false positive KO predictions. The smaller peaks showing in some curves (e.g., the two extra peaks in the blue “1 copy” curve) were found to be due to stretches of intergenic reads that mismapped to KO genes in the database and likely reflect genomic misannotations or pseudogenes. (**b**) The average recall across all simulated 101-bp datasets for identifying reads originating from each KO, ranked from highest to lowest average recall. 95% confidence intervals are shown in green. Recall is calculated for the case where the strain from which the read originated is absent from the database.

As discussed above, a BLAST-based search independently maps each read to the reference database, and in the previous sections, we therefore focused on the ability of such a process to correctly recover the annotation of any single read. In practice, however, the various annotations obtained for the set of reads in a given sample are commonly analyzed jointly, as the overall functional profile of the metagenome is of more interest than the annotation of individual reads. In this context, erroneous annotation of individual reads may be acceptable as long as the combined annotation profile is relatively accurate. The accuracy of this combined profile may be especially important when downstream integrative analyses or pathway-level reconstructions (e.g., [20]) are performed. In this section we accordingly examined the annotation profile obtained for a complete set of reads by aggregating the counts calculated for each KO across all reads in each 101-bp dataset (Table S3; *Methods*). We then compared these aggregated counts to the KO profile of the genome from which each dataset was derived. Since copy numbers are a more intuitive measure than relative abundances, we further translated these aggregated KO counts into copy number estimates by normalizing KO counts by the length of the gene and dividing by the average normalized counts of 15 highly-conserved single-copy genes (*Methods*; [34]). By examining the predicted copy number of each KO in each dataset and comparing it to the actual copy number of the KO in the corresponding genome, we found that direct short read annotation successfully distinguished KOs with different copy numbers (Figure 5a). Predicted copy numbers were distributed normally around the expected value, with greater deviation for higher copy numbers, as expected for a constant error rate per read. This analysis, however, also illustrates the relatively high rate of false predictions, wherein KOs that are in fact absent from the sample are predicted to be present. To determine how accurately the recovered KO abundance profiles reflected the actual KO abundance in each dataset, we calculated the Jensen-Shannon distance between the predicted and actual distributions of KO copy numbers, comparing the performances of the 4 protocols described above. We again found that the *top gene* protocol had the most accurate representation of KO copy numbers, regardless of phylogenetic coverage (Table S3; paired t-test; *Methods*).

Having characterized the mappability of >143 million reads originating from many different genomes and associated with thousands of different orthology groups (KOs), we finally examined the variability in the recall of reads originating from different KOs. Such variability makes some KOs easier to identify than others through short read annotation schemes, potentially biasing the obtained functional profile of a metagenomic sample. As demonstrated in Figure 5b, recall values across KOs are relatively consistent and high for most KOs, with >88% of the KOs in our analysis having >0.85 average recall. Yet a small number of KOs have consistently low recall. To determine whether poorly mapped KOs tend to be associated with specific functions, we examined the KOs in the bottom 5% of recall. We found that the pathways for lysine-, polycyclic aromatic hydrocarbon-, bisphenol-, limonene and pinene-, aminobenzoate-, and ethylbenzene degradation were all enriched in these poorly mapping KOs (hypergeometric test at 0.05 FDR; Table S4). We additionally computed the correlation between the obtained recall values and various gene properties derived from the KEGG database to gain insight into potential determinants of mappability (*Methods*). We found that recall decreases for KOs with higher variability in length and copy number, suggesting as expected that low sequence conservation can lead to lower mappability [35], and for KOs containing genes with high sequence similarity to genes in other KOs (Table S4). This gene-specific variability should be considered when analyzing the functional composition of metagenomes and when performing comparative metagenomic analysis. A list of these poorly mappable KOs, with the average recall and precision obtained, can be found in Table S5.

### Evaluating the impact of annotation protocols on functional profiles obtained for HMP samples

In the analyses above, we have characterized translated-BLAST-based annotation of short metagenomic reads through the use of carefully controlled simulations. Such simulations allow for the precise quantification of the accuracy and error in the annotation process as a function of various biological and technological parameters. In this section, we further explored the impact of the four translated-BLAST based annotation protocols described above on the annotation of real biological samples and examined their impact on the obtained functional profiles. To this end, we used these protocols to re-annotate 15 stool samples from the Human Microbiome Project (HMP) [4], [26] and translated the obtained KO abundance profiles to pathway abundance profiles to determine whether the different protocols led to discrepancies in the perceived pathway-level functional profile of each sample and whether such an effect could mask real biological signals (see *Methods*).

**Figure 6:**
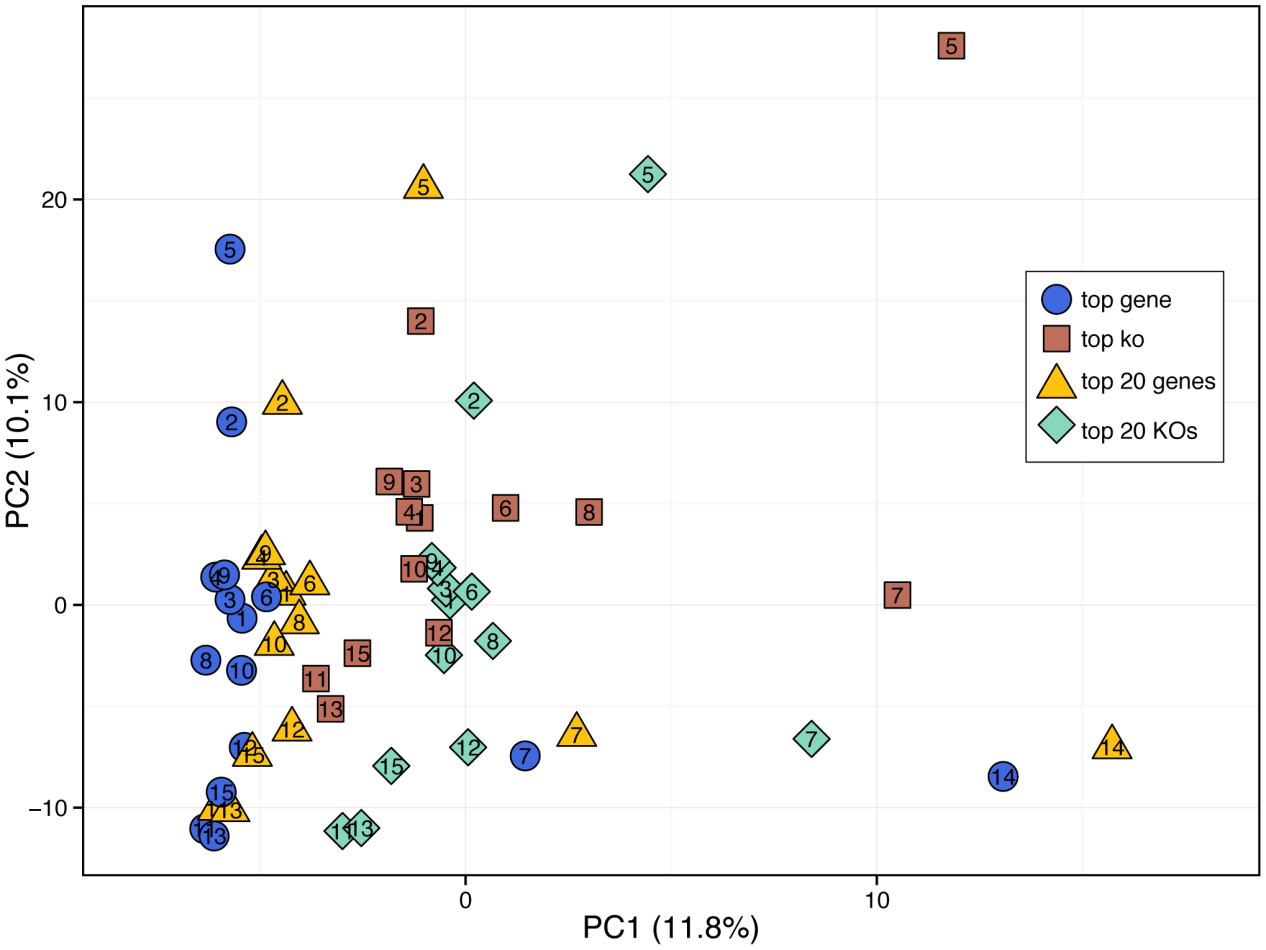
A principal component analysis of the pathway abundance profiles obtained for the 15 analyzed HMP samples and by the four different annotation protocols. HMP samples are numbered from 1 to 15 according to the list that appears in *Methods*. The different protocols are represented by color and shape. Note that two outlier protocols for sample 14 are not shown but were included in the PCA calculation.

We first set out to compare the variation in the functional profile introduced by the selection of a protocol to the natural variation in functional profiles between different samples. We accordingly calculated the Jensen-Shannon distances between the profiles obtained by the different protocols for any given sample and the Jensen-Shannon distances obtained for the various samples by any given protocol. Importantly, we found that the inter-protocol distances are on the same order of magnitude as the inter-sample distances for any one protocol (Table S6). A similar pattern was also observed for the KO abundance profiles (Table S6). Moreover, performing a principal component analysis of the calculated pathway abundance profiles, we found that the first principal component separates samples mostly by annotation protocol and that inter-sample variation is mostly captured only by the second component (Figure 6). This pattern, wherein variation introduced by protocol selection masks true biological variation between samples, is analogous to a batch effect often observed when mixing samples derived by different experimental procedures. Finally, to demonstrate that these apparent protocol-dependent discrepancies introduced consistent biases in pathway abundances and could therefore impact downstream comparative analysis, we examined which pathways appear to be differentially abundant between the profile obtained by one protocol (‘top gene’) and those obtained by other protocols. We found that in fact the vast majority of pathways were differentially abundant between protocols (Wilcoxon-signed rank test at 0.05 FDR; Table S7).

Combined, these findings clearly demonstrate that the choice of functional annotation protocol plays a significant role in analyzing a metagenomic sample and greatly impacts the obtained functional profiles. More importantly, our analysis indicates that inter-protocol variation is comparable to inter-sample variation, suggesting that protocol choice can dramatically impact biological interpretation, potentially obscuring real biological signals and impeding downstream comparative analysis.

### Comparison of alignment strategies

Functional annotation of short metagenomic sequencing reads is most often performed with a translated BLAST search, which aligns a nucleotide sequence to amino acid sequences using all six possible nucleotide reading frames. As discussed above, this alignment strategy sacrifices accuracy in taxonomic labeling of the sequencing read for improved detection of potential protein homology at longer evolutionary distances. While BLAST-based alignments are likely to remain a viable option in coming years, the growth of microbial genome collections, especially in the context of specific environments, has rendered high-quality alignment of sequencing reads directly to genomes an increasingly attractive option for annotating reads. To further explore the tradeoff between identification of function and identification of taxonomy made by these protocols, we compared the performance of translated BLAST to a commonly-used high-quality aligner (BWA [36]), examining the obtained precision and recall of functional annotation using a set of reference genomes at an increasing evolutionary distance. To this end, we re-annotated the set of short reads derived from *S. pneumonia* described above, but with groups of genomes removed from the reference database using a 16S-based distance cutoff (*Methods*, and Figure S3). As expected, we find that at short evolutionary distances, BWA has a marginally higher precision and recall than BLAST for identifying KOs (Figure S4). Importantly, however, recall for BWA approaches zero at longer evolutionary distances and its precision drops dramatically once the number of Bacterial and Archaeal genomes in the reference database is small, while translated BLAST maintains a relatively high recall and precision (Figures S3 and S4).

## Discussion

We have presented a systematic and comprehensive study of BLAST-based functional annotation of short sequencing reads and rigorously characterized annotation protocols commonly used in metagenomic studies. We found that in general such direct annotation of short reads accurately identifies both individual reads and the functional profile of entire samples, but that there is significant variability in annotation accuracy, with many factors impacting the annotation process. For example, the obtained accuracy is affected both by the phylogenetic coverage of the database, with related genomes from the same genus being necessary to accurately annotate most species, and by the phylogeny of the strain in question, with the recall being strongly clade-dependent. Read length was also found to play an important role, with the permissive E-value thresholds appropriate for identifying correct short-read alignments (Text S1 and Figure S2) leading to a decreasing accuracy for longer (>200-bp) reads. Once such longer-read technologies become widely available, new strategies for read-based annotation will be necessary, including length-dependent E-value cutoffs and, ideally, incorporating additional methods, such as contig assembly and gene calling [37]–[39]. In contrast, our analysis demonstrated that typical next-generation sequencing error had little effect on annotation accuracy, probably owing to the nature of translated BLAST searches.

Taken together, our findings highlight the need to design a study-specific pipeline, taking into account the goals of the study and the relative importance of each bias in the annotation process, such as the tradeoff between recall and precision. For example, a comparative metagenomic study that aims to identify differentially abundant genes or pathways in some disease (e.g., Refs. [6], [9], [10], [18]) requires an accurate estimation of gene abundances and may apply a different annotation protocol compared to a study aiming to reconstruct a metagenome-level metabolic network (e.g., [40]) that requires accurate prediction of gene presence. These tradeoffs, and the importance of specific annotation parameters and protocols, may also account for previously reported poor performances of homology-based annotation of short reads in terms of both recall [41] and precision [35]. Our findings suggest that these could be partly rescued by, for example, using a more permissive E-value cutoff to gain a higher recall and using a broader database to gain high precision. Specifically, annotation procedures using a reference database containing only genes associated with known orthology groups (such as COG), rather than representatives of all genes, effectively use a ‘*top KO’* approach which decreases precision.

Importantly, while the analysis presented here relied specifically on KEGG genome annotations, the conclusions are likely applicable to any gene orthology-based database. More generally, the functional annotation protocols evaluated here are clearly based on the conjecture that sequence homology is an indicator of shared function. This orthology–function conjecture has been shown to hold as a general trend, but is also known to be false in at least some cases [42]. Nonetheless, as demonstrated above, the functional profiles estimated by such homology-based annotation protocols clearly successfully reconstruct the overall functional profile of the sampled community.

Notably, successors to short-read sequencing technologies, such as long-read sequencing and single-cell genomics, are on the horizon. When such technologies become standard components of microbiome experiments, translated BLAST-based functional annotation may no longer be the best option for estimating the functional profile of a community. Translated BLAST alignments also require relatively long runtimes compared to approaches like BWA [36] or Bowtie [43] (though many specialized, efficient implementations of BLAST have been recently introduced [44], [45]). These newer technologies, however, are still in their early stages and at least several years will be required before they are widely adopted. Furthermore, strategies for mapping short metagenomic reads to reference genomes from highly-related strains (e.g., BWA [36], Bowtie [43]) require near-complete genome coverage of a microbial community to provide a reliable community-level functional description. While it is likely that the human microbiome will be relatively well covered within the next decade, highly diverse and open environments such as the ocean may never have complete genome catalogs. Translated BLAST-based approaches, and more generally, homology-based annotation protocols, therefore still represent a viable option for functional characterization of metagenomic samples for years to come.

Overall, our findings offer an improved understanding of homology-based annotation capabilities and delineate the boundaries of metagenomic functional annotation practices. The clade-dependent accuracy of the obtained annotation we observed above highlights the pressing need to extend the collection of reference genomes available and to capture a wider diversity of gene orthology groups through efforts such as the Genomic Encyclopaedia of Bacteria and Archaea (GEBA) Project [46] and especially the Microbial Dark Matter (MDM) project [47]. The results and methodologies of our study can be used to pinpoint particular phylogenetic branches for additional sequencing, such as branches with consistently poor functional annotation that suggest sparse coverage of representative sequences. Our findings indeed demonstrate that including a diverse representation of genera should be a priority for sequencing programs. Clearly, this need for diversity should be balanced with efforts to reduce the complexity of the database (e.g., using representative sequences as in the Pfam database [48]) and with improved characterization of the link between sequence homology and shared function [42]. Ultimately, however such combined efforts, aiming to both improve annotation practices and expand reference microbial databases, will allow us to move toward a more accurate annotation of metagenomic data and a more complete understanding of the functional capacities encoded by microbial communities.

## Methods

### Simulating sequencing reads

Simulated sequencing reads were generated by sliding a fixed-size window across complete genomes obtained from the KEGG database (KEGG v. 63; July 2^nd^, 2012 weekly release) [12]. The genome of *Streptococcus pneumoniae* ATCC 700669 (KEGG code *sne*), was used to test the effects of read length and sequencing error. Specifically, to test the effect of read length, 6 datasets of simulated reads were created, including *every* possible read of lengths 75, 101, 150, 200, 300, and 400 bp. The 101-bp dataset was then used as the basis for simulating reads with sequencing error rates of 0.15%, 1.5%, and 3%. To simulate this next-generation sequencing error, a position-dependent error profile created with Ibis [49] from an Illumina sequencing run with a total error rate of 0.15% was applied and uniformly magnified to achieve the desired error rate. To study the effect of phylogeny, simulated sequencing reads were further generated from 167 bacterial and archaeal genomes included in the tree of life constructed by Ref. [50] and from two additional human-commensal genomes of *E. coli* and *B. fragilis*. See Table S1 for a complete list of genomes used in this analysis. Organisms from the tree of life of Ref. [50] were mapped to KEGG organisms first by NCBI TaxID, and then by matching the strain name if the NCBI TaxID did not match. Species for which more than one KEGG organism existed were mapped to an arbitrary strain from that species. One species, *Gemmata obscuriglobus*, did not have a representative strain in KEGG and was not included in the analysis. Sets of simulated reads consisting of *every* possible 101-bp read were generated for the two human-commensal genomes and for one organism from each of the 23 Phyla present in the tree (resulting in >100-fold coverage). Alpha-, beta-, gamma-, delta-, and epsilonproteobacteria were considered separate phyla in the analysis. For the remaining 144 genomes, simulated reads were generated representing every 10^th^ possible sequencing read by moving a sliding window over the genome at 10 bp jumps (resulting in a >10-fold coverage). A complete list of the 178 datasets of simulated reads generated for this analysis is provided in Table S2.

### Mapping and annotating reads

To annotate the simulated reads, each sequencing read from each dataset was aligned to a custom peptide database containing the peptide sequences from all annotated KEGG organisms (KEGG v. 63; July 2^nd^, 2012 weekly release [12]). To this end, Translated Query-Protein Subject BLAST (blastx) v. 2.2.25+, with standard parameters and accepting all matches with an E-value<1 (as done by the HMP [4], [20]) was used (average execution time 7.6098±0.76247 s/read). To test the effect of low-complexity sequence filtering on the obtained alignments, identical searches were performed with SEG filtering [51] disabled for all error-free *S. pneumoniae* datasets (see Text S1). To examine the impact of the phylogenetic coverage of the database, additional BLAST results were simulated by removing alignments to the correct strain, species, or genus from the original set of BLAST alignments. This is mathematically equivalent to a scenario where database sequences from the corresponding strain, species, or genus are replaced with non-matching sequences of identical lengths, thereby preserving all database size-based metrics (e.g. E-value).

Once reads were mapped to the KEGG database, each read was annotated according to the KEGG Orthology groups (KOs) associated with the identified alignments. As described in the main text, four different protocols were applied to determine which of the potentially many alignments found would be used to annotate the read: (i) *‘Top gene’* – the read was annotated based on the top-scoring alignment (i.e., the alignment(s) with the smallest E-value), regardless of whether this alignment was associated with a KO or not. If several alignments were tied for the top score, all were used. If the top scoring alignment(s) was not associated with any KO, the read was annotated has having *no result*. (ii) *‘Top KO’* – the read was annotated based on the top scoring alignment(s) that was associated with a KO. Potentially better scoring alignments that were not associated with a KO were ignored. (iii) *‘Top 20 genes’* – the read was annotated based on the top 20 scoring alignments (i.e., the up-to 20 alignments with the smallest E-values), regardless of whether these alignments were associated with a KO or not. (iv) *‘Top 20 KOs’* – the read was annotated based on all alignments that were associated with a KO among the top 20 scoring alignments. In case of top scoring ties or in protocols where multiple alignments were considered (i.e., *top 20 genes* or *top 20 KOs*), the read was annotated based on all the selected alignments weighted by their P-value using the following formula: 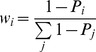
(1)or
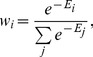
(2)where 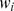 is the weight, 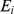 the E-value for annotation *i*, and 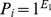 is the corresponding P-value. As the E-value denotes the expected number of matches for an alignment of a given score in the given database, and the P-value denotes the probability of finding at least one match with at least this score in the database, considering the P-value in this manner thus weights the matches by their relative probabilities of occurring by chance. While this weighting scheme was employed by the HMP, it was found that the functional form of the weighting scheme and the alignment parameter used (E-value, bit-score) had little effect on the annotations [20]. If the collection of alignments were associated with more than one KO, the read would therefore be annotated with multiple KOs, each given a fractional count that summed to unity. Notably, cases in which alignments not associated with a KO were considered (e.g., in the *top 20 genes* protocol), these alignments clearly did not contribute to the set of KOs with which the read was annotated but were still considered in the weighting scheme, thereby lowering the fractional counts given to the other KO alignments.

### Evaluating obtained annotations

To evaluate the accuracy of the annotation process, the annotations obtained for each read were compared to the annotations associated with the genomic region from which the read originated. Specifically, each read could have originated from a *KO gene* (a gene associated with a KO annotation), a *non-KO gene* (a gene with no known KO annotation), or from an *intergenic* region. Reads that originated from multiple genomic categories (e.g., reads that span both a gene and an intergenic region) were excluded from the analyses as the ‘correct’ annotation was not well defined. Repeating the analysis with such multiple-category reads included by weighing the categories with the relative coverage within each read did not qualitatively change the results reported in the main text (Table S3; and see also Text S1 and Figure S5 for an analysis of the mappability of partially overlapping reads). Considering these categories, the set of alignments and annotations obtained for each read could then be classified (Figure 1a). Specifically, each alignment obtained for a read originating from a KO gene could be classified as (a) correct gene and correct KO, (b) incorrect gene but correct KO, (c) incorrect gene and incorrect KO, or (d) incorrect gene that is not associated with a KO. Similarly, each alignment obtained for a read originating from a non-KO gene could be classified as (a) correct gene that is, as expected, not associated with a KO, (b) incorrect gene that is not associated with a KO, or (c) incorrect gene and incorrect KO. Finally, an alignment obtained for a read originating from an intergenic region could be classified as (a) incorrect gene and incorrect KO, or (b) incorrect gene that is not associated with a KO. Reads for which no alignment was found by blastx were classified as *‘no result’*. For alignment ties or in protocols were multiple alignments could be considered (e.g., top 20 genes), the classifications of all alignments considered were weighted as described above. To evaluate the performance of the annotation process in each dataset, the overall recall and precision of the obtained annotations were calculated. When comparing annotation performance across read lengths, all reads were included in the analysis, as multiple-category reads accounted for almost half the reads for longer sequencing reads.

### Evaluating the annotation profile for a complete sets of reads

When evaluating the accuracy of the annotation profile obtained for a complete set of reads (e.g., all reads simulated from a specific genome), the total count for each KO was calculated as the sum of all counts toward this KO obtained across all the reads included in this dataset. KO counts were then normalized by the length of the gene to correct for length-bias in read counts. To simulate the case where the actual gene from which the read originated was not known, gene lengths were approximated by the length of the gene to which the read was aligned. For comparison, KOs were alternatively normalized by the average length of all the genes associated with the KO. This scheme, however, resulted in reduced accuracy across all protocols and at all levels of phylogenetic coverage (Table S3). Finally, to estimate the copy number of each KO, these normalized counts were divided by the mean count over a set of 15 ribosomal genes found with 1 copy across all bacteria and archaea in the KEGG database [34]. To estimate how well the predicted KO copy numbers in each sample reflected the actual KO copy numbers, the square root of the Jensen-Shannon divergence (JSD), the Jensen-Shannon distance, between the calculated and actual KO copy numbers was calculated. To gain insight into potential determinants of mappability, we further computed the correlation between the recall values obtained and the average, standard deviation, and coefficient of deviation of the gene lengths for each KO, the number of genes in each KO, the number of genomes covered by each KO, and the mean and standard deviation of genes per genome. Additionally, we computed the correlation between the recall values and the fraction of matches to genes from different KOs and the percent-identity of the highest match to a gene from a different KO in the KEGG Orthology SSDB using a gene chosen randomly for each KO from the genomes used in this study [12].

### Evaluating annotation profiles obtained for HMP stool samples

Human sequence-filtered and quality-filtered, Illumina shotgun sequences were downloaded from the Human Microbiome Project Data Analysis Coordination Center (http://HMPDACC.org) for 15 stool samples from the original HMP cohort (SRS011084, SRS024075, SRS024388, SRS011239, SRS020233, SRS011302, SRS049900, SRS011271, SRS015190, SRS011529, SRS016989, SRS011134, SRS058770, SRS023971, SRS021484). Sequencing reads were aligned to the same custom peptide database as the simulated reads using mBLAST (mblastx) [44], a multicore, shared memory implementation of the Translated Query-Protein Subject BLAST (blastx), with standard parameters and accepting all matches with an E-value<1 (average execution time 0.6±0.8 ms/read). KO abundances were computed using each of the four protocols, as outlined above. Pathway abundances were calculated by summing the abundances of all KOs associated with each pathway, and the pathway abundances for each sample were normalized by the total pathway count. Only pathways with differences in ≥10 samples were tested for differential abundance.

### Comparison of functional annotations derived with BWA to translated BLAST alignments

Custom reference databases for BWA (nucleotide sequences) and BLAST (peptide sequences) were constructed from gene sequences from all annotated KEGG organisms (KEGG v. 63; July 2^nd^, 2012 weekly release [12]) that were at an evolutionary distance greater than some cutoff from *Streptococcus pneumoniae* ATCC 700669 (KEGG code *sne*). To determine these evolutionary distances, we used the protocol employed in Refs. [52], [53]. Briefly, 16S rRNA gene sequences were extracted from all Archaea and Bacteria in KEGG. If an Archaeon or Bacterium had no annotated 16S rRNA genes, then 16S rRNA sequences were obtained from the same taxa in the Integrated Microbial Genomes (IMG) database, v. 4.0 [54], using the corresponding NCBI Taxon ID. 76 Bacteria and Archaea from KEGG had no 16S gene annotated in either KEGG or the IMG and were excluded from the analysis. 16S rRNA genes were then aligned to the Greengenes core set using PyNAST v. 0.1 [55]. Distances between all pairs of aligned 16S rRNA sequences were computed using Clearcut v. 1.0.9 [56]. The evolutionary distance from the *S. pneumoniae* ‘*sne*’ genome was then calculated as the mean distance between all *sne* 16S rRNA genes and all 16S rRNA genes from a given genome. BWA databases were constructed at evolutionary distances of 0.0, 0.05, 0.125, 0.2, 0.275, 0.35, 0.425, 0.5, and 0.575 (Figure S3). At short evolutionary distances (0.0, 0.05, 0.125), BLAST matches were found by removing alignments to genomes at evolutionary distances below the cutoff (or to genomes that had been excluded from the analysis) from the alignment to all protein-coding genes from annotated organisms, as described above. For evolutionary distances longer than 0.125, new BLAST databases were constructed to avoid skewing the E-value metrics by changing the database size. Similar to previous simulations, all possible contiguous 101-bp reads from the *S. pneumoniae* ‘*sne*’ genome were aligned to each database. Translated Query-Protein Subject BLAST (blastx) alignments were performed as done previously. BWA alignments were performed using BWA-MEM version 0.7.8 [36], taking the top alignment. Functional annotation was performed as described above, using the ‘top gene’ approach for the translated BLAST alignments.

## Supporting Information

Figure S1 **The precision (a, circles) and recall (b, squares) for identifying KEGG orthology groups from short sequencing reads using different E-value cutoffs as a function of read length.**
(TIF)

Figure S2 **The distribution of E-values from translated BLAST searches of simulated short sequencing reads against all annotated peptides from the KEGG database.** The distributions obtained for the (**a**) 101-bp and (**b**) 400-bp datasets derived from the *S. pneumoniae* genome are shown. The different colors represent the various categories of reads and their annotation as in Figure 1. The bimodal distribution seen in (**b**) is due to the use of a low-complexity filter in the translated BLAST search (See Text S1).
(TIF)

Figure S3 **The number of available bacterial and archaeal genomes as a function of 16S distance from** ***Streptococcus pneumoniae*** **ATCC 700669 (KEGG code** ***sne*** **), represented as a histogram.** Vertical dashed lines highlight the evolutionary distance cutoffs used to construct reference genome databases for comparing BWA and BLAST alignments (see *Methods* and also Figure S4).
(TIF)

Figure S4 **The precision (a, circles) and recall (b, squares) for BWA- (white) and translated BLAST- (blue) based functional annotation of the** ***Streptococcus pneumoniae*** **ATCC 700669 (KEGG code** ***sne*** **) genome as a function of the minimal evolutionary distance to genomes included in the reference database.**
(TIF)

Figure S5 **The mappability of reads that only partially overlap a KO gene through a translated BLAST search.** The probability of such overlapping reads to correctly map to the KO of origin, vs. the probability to erroneously map to a gene from an incorrect KO, a non-KO gene, or to have no result is shown as a function of the number of bases the read overlaps with the gene. Probabilities are averaged across all 101-bp datasets, with error bars representing 95% confidence intervals.
(TIF)

Table S1 **Summary of all genomes used in this study.**
(DOCX)

Table S2 **Summary of all datasets generated for this study.**
(DOCX)

Table S3 **Summary of all results obtained for each simulated dataset.**
(XLS)

Table S4 **Correlation of various KO properties with KO average recall values, and pathways enriched (FDR<0.05) among the KOs in the bottom 5% of recall.**
(XLS)

Table S5 **The average recall and precision obtained per KEGG Orthology Group (KO). Poorly mapping genes (Recall<85%) are highlighted in red.**
(XLSX)

Table S6 **The Jensen-Shannon distance between KO or pathway abundance profiles for different samples and for the same sample annotated with different annotation protocols.**
(XLS)

Table S7 **Pathways and KOs enriched between HMP stool samples annotated with different functional annotation protocols.** A ‘1’ indicates enrichment (FDR<0.05) and ‘0’ indicates no enrichment.
(XLS)

Text S1 **An analysis of E-value cutoff schemes and the effect of low-complexity filtering on typical E-value distributions, and an analysis of reads originating from multiple genomic regions.**
(DOCX)
