# Supplementary figures and images for: Comparative Analysis of Functional Metagenomic Annotation and the Mappability of Short Reads
Source: PLoS One. 2014 Aug 22;9(8):e105776. doi: 10.1371/journal.pone.0105776 (PMC4141809; doi:10.1371/journal.pone.0105776)

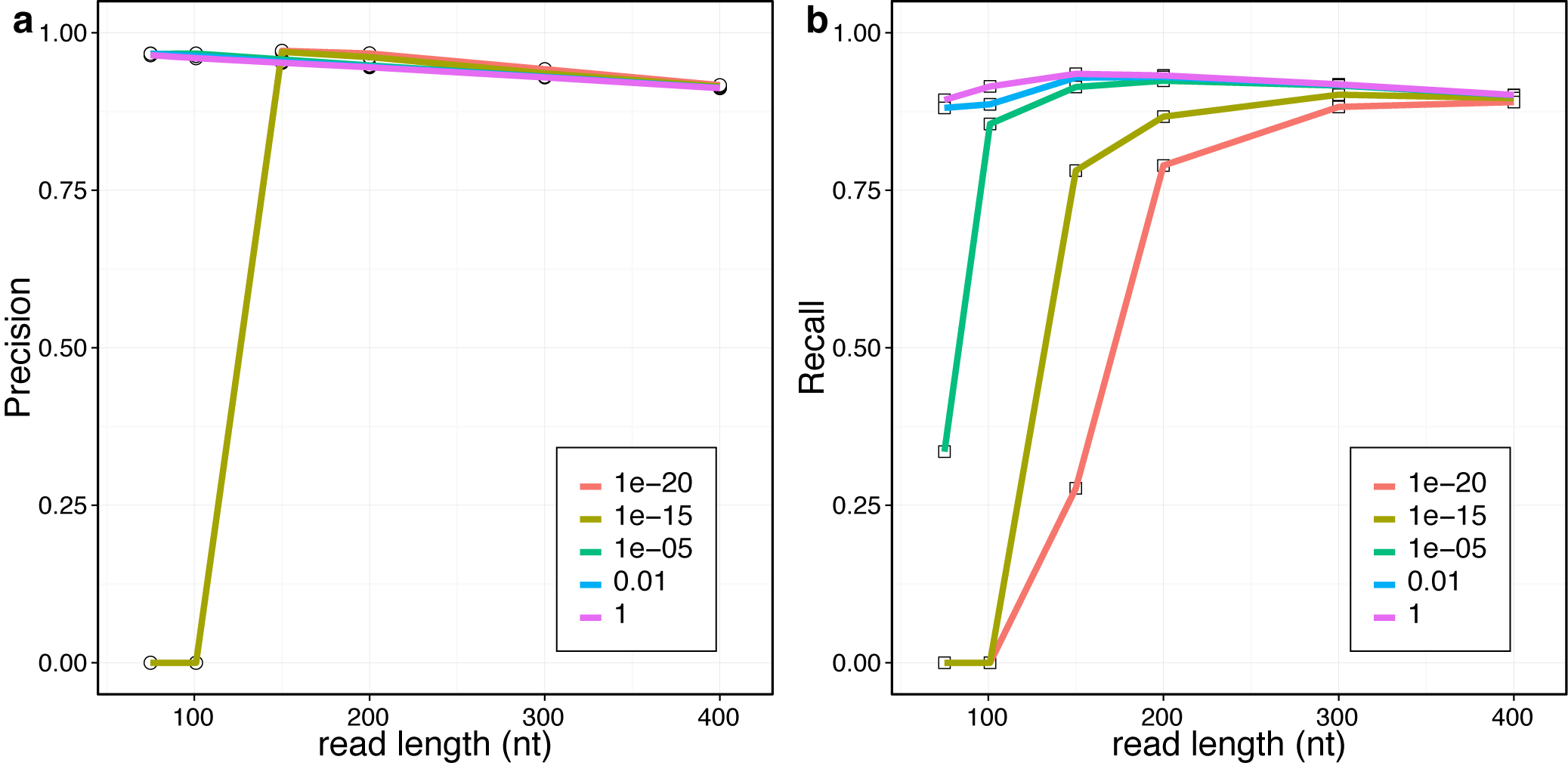

Supplement: Figure S1 — The precision (a, circles) and recall (b, squares) for identifying KEGG orthology groups from short sequencing reads using different E-value cutoffs as a function of read length. (TIF) [file pone.0105776.s001.tif]

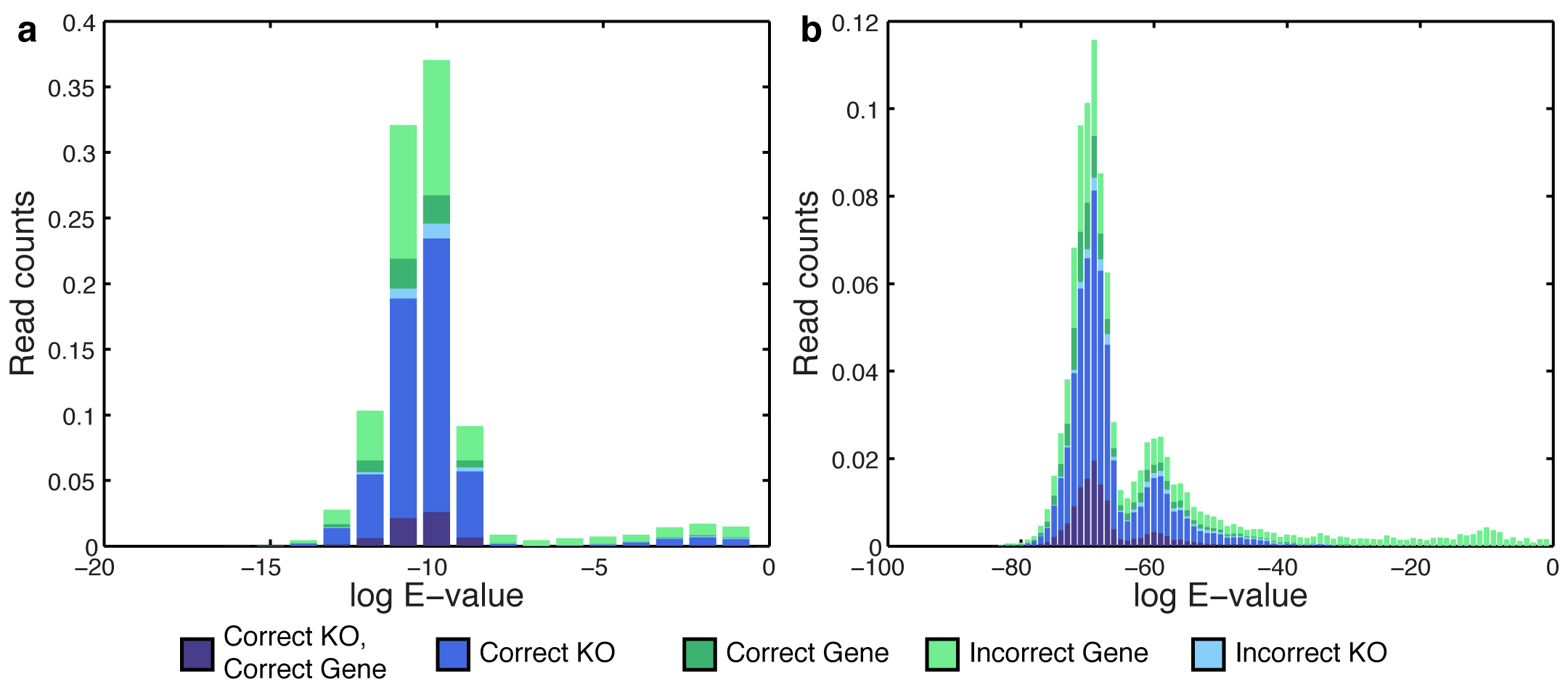

Supplement: Figure S2 — The distribution of E-values from translated BLAST searches of simulated short sequencing reads against all annotated peptides from the KEGG database. The distributions obtained for the (a) 101-bp and (b) 400-bp datasets derived from the S. pneumoniae genome are shown. The different colors represent the various categories of reads and their annotation as in Figure 1. The bimodal distribution seen in (b) is due to the use of a low-complexity filter in the translated BLAST search (See Text S1). (TIF) [file pone.0105776.s002.tif]

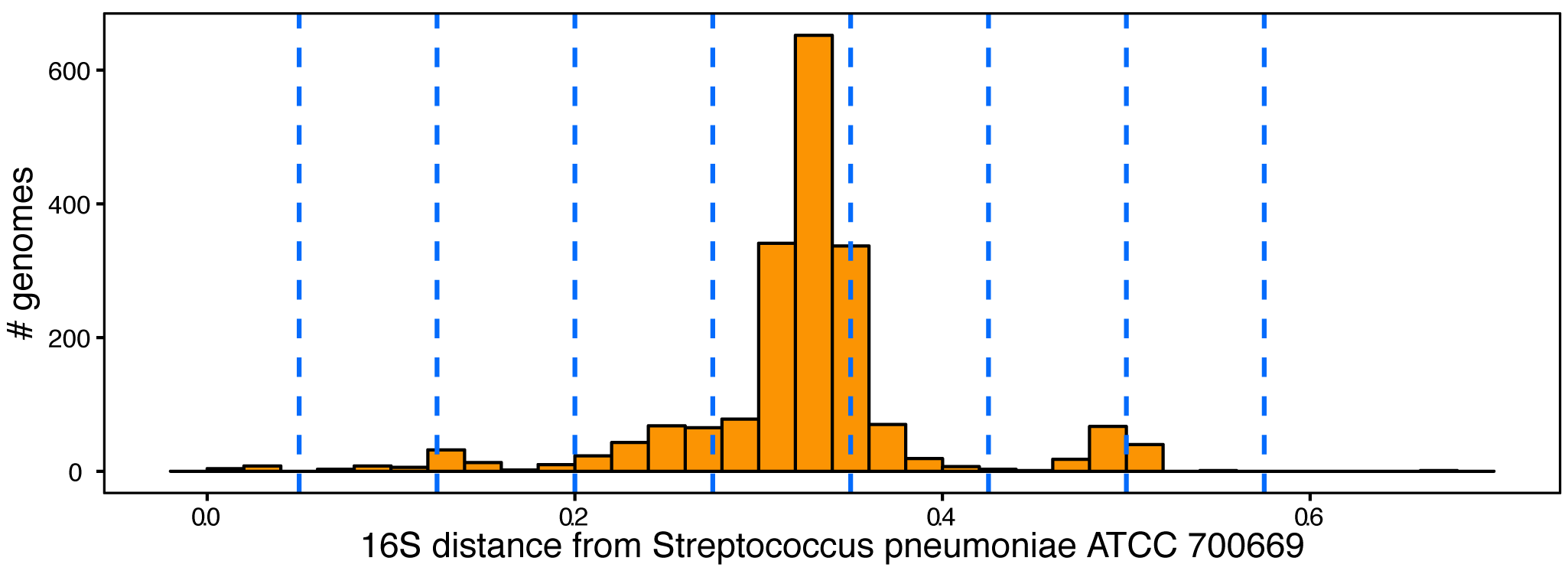

Supplement: Figure S3 — The number of available bacterial and archaeal genomes as a function of 16S distance from Streptococcus pneumoniae ATCC 700669 (KEGG code sne ), represented as a histogram. Vertical dashed lines highlight the evolutionary distance cutoffs used to construct reference genome databases for comparing BWA and BLAST alignments (see Methods and also Figure S4). (TIF) [file pone.0105776.s003.tif]

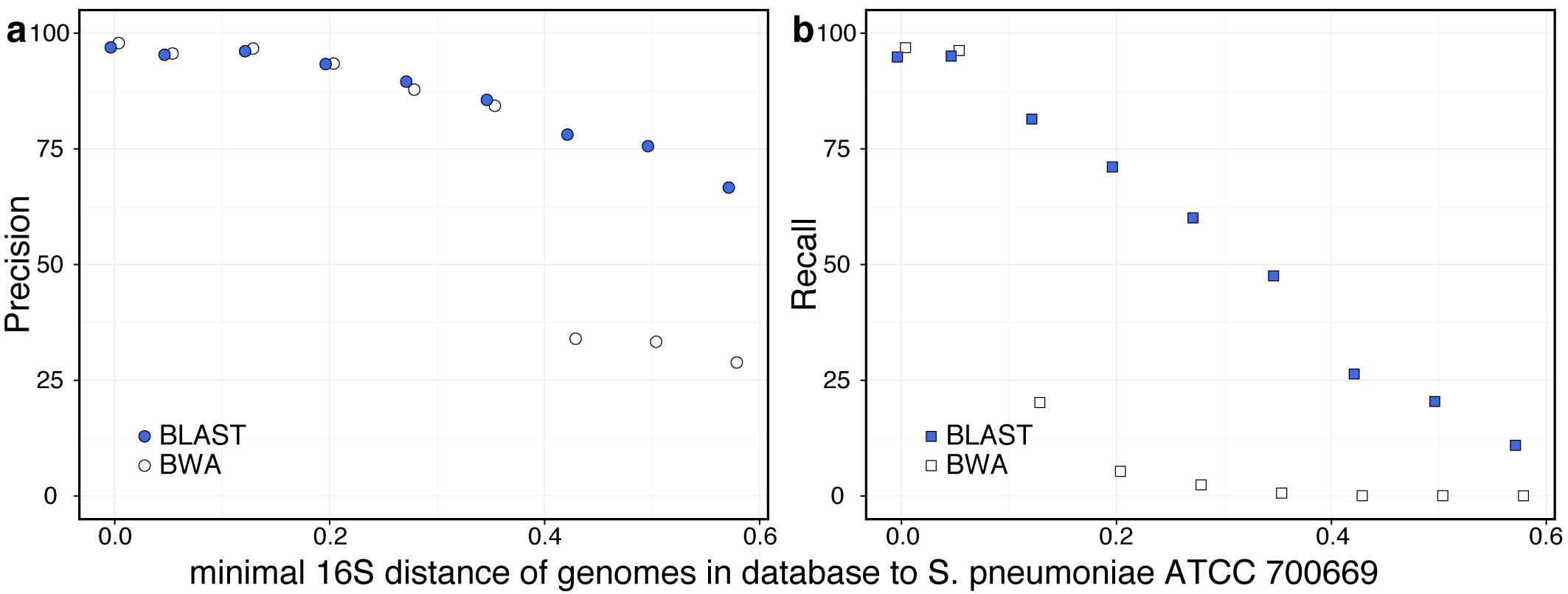

Supplement: Figure S4 — The precision (a, circles) and recall (b, squares) for BWA- (white) and translated BLAST- (blue) based functional annotation of the Streptococcus pneumoniae ATCC 700669 (KEGG code sne ) genome as a function of the minimal evolutionary distance to genomes included in the reference database. (TIF) [file pone.0105776.s004.tif]

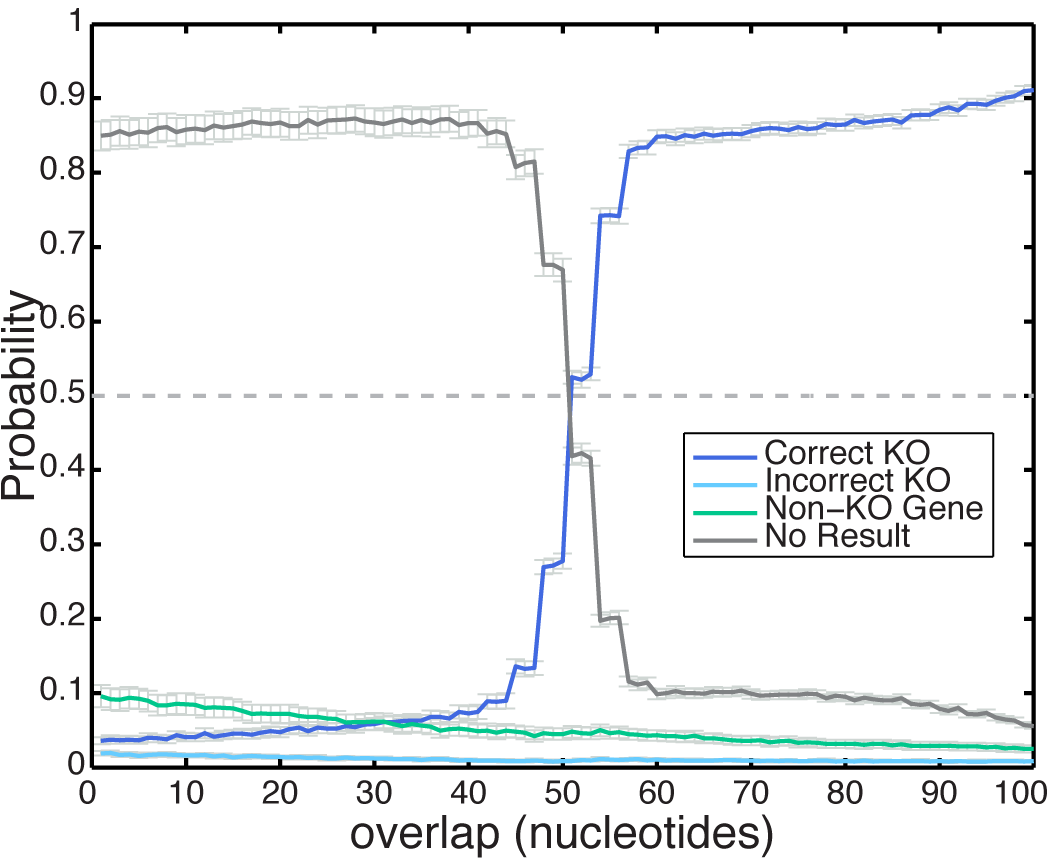

Supplement: Figure S5 — The mappability of reads that only partially overlap a KO gene through a translated BLAST search. The probability of such overlapping reads to correctly map to the KO of origin, vs. the probability to erroneously map to a gene from an incorrect KO, a non-KO gene, or to have no result is shown as a function of the number of bases the read overlaps with the gene. Probabilities are averaged across all 101-bp datasets, with error bars representing 95% confidence intervals. (TIF) [file pone.0105776.s005.tif]
