# Supplementary material for: Comparative Analysis of Functional Metagenomic Annotation and the Mappability of Short Reads
Source: PLoS One. 2014 Aug 22;9(8):e105776. doi: 10.1371/journal.pone.0105776 (PMC4141809; doi:10.1371/journal.pone.0105776)
Supplement: Table S1 — Summary of all genomes used in this study. (DOCX) [file pone.0105776.s006.docx]

**Table S1:** Summary of all genomes used in this study.

| KEGG ID | Phylum | Genus | Species | Strain | # genomes from Genus in KEGG | # genomes from Species in KEGG | # Protein Genes | # Protein KOs |
| --- | --- | --- | --- | --- | --- | --- | --- | --- |
| sne | Firmicutes | Streptococcus | pneumoniae | ATCC 700669 (serotype 23F ST81 lineage) | 79 | 17 | 1990 | 1156 |
| ecl | Gammaproteobacteria | Escherichia | coli | C ATCC 8739 | 53 | 51 | 4199 | 2922 |
| bfg | Bacteroidetes | Bacteroides | fragilis | 638R | 7 | 3 | 4290 | 1309 |
| aae | Aquificae | Aquifex | aeolicus | - | 1 | 1 | 1560 | 1033 |
| aca | Acidobacteria | Acidobacterium | capsulatum | - | 1 | 1 | 3377 | 1407 |
| bba | Deltaproteobacteria | Bdellovibrio | bacteriovorus | - | 1 | 1 | 3586 | 1452 |
| bbz | Spirochaetes | Borrelia | burgdorferi | ZS7 | 12 | 2 | 1239 | 528 |
| bln | Actinobacteria | Bifidobacterium | longum | subsp. infantis ATCC 15697 (JGI) | 23 | 9 | 2416 | 1085 |
| bme | Alphaproteobacteria | Brucella | melitensis | bv. 1 16M | 18 | 6 | 3198 | 2011 |
| bpe | Betaproteobacteria | Bordetella | pertussis | Tohama I | 6 | 2 | 3436 | 1905 |
| bsu | Firmicutes | Bacillus | subtilis | - | 53 | 5 | 4176 | 2197 |
| cte | Chlorobi | Chlorobaculum | tepidum | - | 2 | 1 | 2245 | 1108 |
| ctr | Chlamydiae | Chlamydia | trachomatis | D/UW-3/CX | 19 | 18 | 895 | 553 |
| det | Chloroflexi | Dehalococcoides | ethenogenes | - | 5 | 1 | 1580 | 764 |
| dra | Deinococcus-Thermus | Deinococcus | radiodurans | - | 6 | 1 | 3167 | 1437 |
| ecf | Gammaproteobacteria | Escherichia | coli | O157:H7 EC4115 (EHEC) | 53 | 51 | 5477 | 2999 |
| fnu | Fusobacteria | Fusobacterium | nucleatum | - | 1 | 1 | 2063 | 1148 |
| fsu | Fibrobacteres | Fibrobacter | succinogenes | - | 2 | 2 | 3085 | 1077 |
| hpy | Epsilonproteobacteria | Helicobacter | pylori | 26695 | 45 | 37 | 1573 | 979 |
| mmd | Euryarchaeota | Methanococcus | maripaludis | X1 | 8 | 5 | 1848 | 931 |
| neq | Nanoarchaeota | Nanoarchaeum | equitans | - | 1 | 1 | 540 | 291 |
| pgt | Bacteroidetes | Porphyromonas | gingivalis | TDC60 | 4 | 3 | 2217 | 845 |
| rba | Planctomycetes | Rhodopirellula | baltica | - | 1 | 1 | 7325 | 1755 |
| sso | Crenarchaeota | Sulfolobus | solfataricus | P2 | 13 | 2 | 2978 | 1445 |
| syc | Cyanobacteria | Synechococcus | elongatus | PCC6301 | 11 | 2 | 2523 | 1337 |
| tma | Thermotogae | Thermotoga | maritima | - | 7 | 1 | 1858 | 1196 |
| afu | Euryarchaeota | Archaeoglobus | fulgidus | - | 3 | 1 | 2420 | 1236 |
| ana | Cyanobacteria | Anabaena | sp. | PCC7120 | 3 | 1 | 6129 | 2122 |
| ape | Crenarchaeota | Aeropyrum | pernix | - | 1 | 1 | 1700 | 903 |
| atu | Alphaproteobacteria | Agrobacterium | tumefaciens | C58 | 4 | 1 | 5355 | 2685 |
| atu | Alphaproteobacteria | Agrobacterium | tumefaciens | C58 | 4 | 1 | 5355 | 2685 |
| bab | Gammaproteobacteria | Buchnera | aphidicola | Bp | 12 | 12 | 507 | 484 |
| bas | Gammaproteobacteria | Buchnera | aphidicola | Sg | 12 | 12 | 546 | 528 |
| bat | Firmicutes | Bacillus | anthracis | Sterne | 53 | 6 | 5289 | 2363 |
| bbr | Betaproteobacteria | Bordetella | bronchiseptica | - | 6 | 1 | 4994 | 2499 |
| bca | Firmicutes | Bacillus | cereus | ATCC 10987 | 53 | 12 | 5843 | 2392 |
| bce | Firmicutes | Bacillus | cereus | ATCC 14579 | 53 | 12 | 5255 | 2439 |
| bfl | Gammaproteobacteria | Candidatus Blochmannia | floridanus | - | 3 | 1 | 583 | 563 |
| bha | Firmicutes | Bacillus | halodurans | - | 53 | 1 | 4065 | 2185 |
| bju | Alphaproteobacteria | Bradyrhizobium | japonicum | USDA 6 | 5 | 2 | 8826 | 2868 |
| bpa | Betaproteobacteria | Bordetella | parapertussis | - | 6 | 1 | 4185 | 2240 |
| bsv | Alphaproteobacteria | Brucella | suis | VBI22 | 18 | 4 | 3270 | 1626 |
| bth | Bacteroidetes | Bacteroides | thetaiotaomicron | - | 7 | 1 | 4816 | 1365 |
| buc | Gammaproteobacteria | Buchnera | aphidicola | APS | 12 | 12 | 574 | 551 |
| cae | Firmicutes | Clostridium | acetobutylicum | DSM 1731 | 39 | 3 | 3922 | 1605 |
| cbu | Gammaproteobacteria | Coxiella | burnetii | RSA 493 | 5 | 5 | 1847 | 961 |
| cca | Chlamydiae | Chlamydophila | caviae | - | 15 | 1 | 1005 | 572 |
| ccr | Alphaproteobacteria | Caulobacter | crescentus | CB15 | 4 | 2 | 3737 | 1798 |
| cdw | Actinobacteria | Corynebacterium | diphtheriae | PW8 | 39 | 13 | 2322 | 1107 |
| cef | Actinobacteria | Corynebacterium | efficiens | - | 39 | 1 | 2994 | 1318 |
| cgb | Actinobacteria | Corynebacterium | glutamicum | ATCC 13032 (Bielefeld) | 39 | 3 | 3057 | 1357 |
| cgt | Actinobacteria | Corynebacterium | glutamicum | R | 39 | 3 | 3080 | 1421 |
| cju | Epsilonproteobacteria | Campylobacter | jejuni | subsp. jejuni 81116 | 14 | 9 | 1626 | 1071 |
| cmu | Chlamydiae | Chlamydia | muridarum | - | 19 | 1 | 910 | 549 |
| cpa | Chlamydiae | Chlamydophila | pneumoniae | AR39 | 15 | 5 | 1112 | 562 |
| cpe | Firmicutes | Clostridium | perfringens | 13 | 39 | 3 | 2723 | 1359 |
| cpj | Chlamydiae | Chlamydophila | pneumoniae | J138 | 15 | 5 | 1069 | 568 |
| cpn | Chlamydiae | Chlamydophila | pneumoniae | CWL029 | 15 | 5 | 1052 | 564 |
| cpt | Chlamydiae | Chlamydophila | pneumoniae | TW183 | 15 | 5 | 1113 | 567 |
| ctc | Firmicutes | Clostridium | tetani | E88 | 39 | 1 | 2439 | 1311 |
| cvi | Betaproteobacteria | Chromobacterium | violaceum | - | 1 | 1 | 4405 | 2282 |
| dvl | Deltaproteobacteria | Desulfovibrio | vulgaris | DP4 | 11 | 4 | 3091 | 1559 |
| ecc | Gammaproteobacteria | Escherichia | coli | O6:K2:H1 CFT073 (UPEC) | 53 | 51 | 5369 | 2972 |
| ece | Gammaproteobacteria | Escherichia | coli | O157:H7 EDL933 (EHEC) | 53 | 51 | 5397 | 3174 |
| eco | Gammaproteobacteria | Escherichia | coli | K-12 MG1655 | 53 | 51 | 4146 | 2907 |
| efa | Firmicutes | Enterococcus | faecalis | V583 | 5 | 3 | 3264 | 1432 |
| gsu | Deltaproteobacteria | Geobacter | sulfurreducens | PCA | 9 | 2 | 3428 | 1566 |
| gvi | Cyanobacteria | Gloeobacter | violaceus | - | 1 | 1 | 4430 | 1588 |
| hal | Euryarchaeota | Halobacterium | sp. | NRC-1 | 2 | 1 | 2622 | 1154 |
| hdu | Gammaproteobacteria | Haemophilus | ducreyi | - | 14 | 1 | 1717 | 1113 |
| hhe | Epsilonproteobacteria | Helicobacter | hepaticus | - | 45 | 1 | 1876 | 989 |
| hiz | Gammaproteobacteria | Haemophilus | influenzae | R2866 | 14 | 9 | 1795 | 1262 |
| hpj | Epsilonproteobacteria | Helicobacter | pylori | J99 | 45 | 37 | 1488 | 945 |
| lic | Spirochaetes | Leptospira | interrogans | serovar Copenhageni | 7 | 3 | 3667 | 1214 |
| lil | Spirochaetes | Leptospira | interrogans | serovar Lai 56601 | 7 | 3 | 3702 | 1259 |
| lin | Firmicutes | Listeria | innocua | - | 17 | 1 | 3043 | 1581 |
| ljo | Firmicutes | Lactobacillus | johnsonii | NCC 533 | 40 | 3 | 1821 | 926 |
| llm | Firmicutes | Lactococcus | lactis | subsp. cremoris MG1363 | 9 | 7 | 2434 | 1257 |
| lmf | Firmicutes | Listeria | monocytogenes | F2365 | 17 | 13 | 2821 | 1541 |
| lmo | Firmicutes | Listeria | monocytogenes | EGD-e | 17 | 13 | 2846 | 1589 |
| lpj | Firmicutes | Lactobacillus | plantarum | JDM1 | 40 | 3 | 2948 | 1494 |
| mac | Euryarchaeota | Methanosarcina | acetivorans | - | 3 | 1 | 4540 | 1700 |
| mbm | Actinobacteria | Mycobacterium | bovis | BCG Mexico | 36 | 4 | 3952 | 1445 |
| mga | Firmicutes | Mycoplasma | gallisepticum | R(low) | 38 | 3 | 763 | 388 |
| mge | Firmicutes | Mycoplasma | genitalium | - | 38 | 1 | 475 | 339 |
| mja | Euryarchaeota | Methanocaldococcus | jannaschii | - | 5 | 1 | 1771 | 1018 |
| mka | Euryarchaeota | Methanopyrus | kandleri | - | 1 | 1 | 1687 | 842 |
| mlb | Actinobacteria | Mycobacterium | leprae | Br4923 | 36 | 2 | 1604 | 894 |
| mlo | Alphaproteobacteria | Mesorhizobium | loti | - | 4 | 1 | 7272 | 3115 |
| mma | Euryarchaeota | Methanosarcina | mazei | - | 3 | 1 | 3368 | 1443 |
| mmo | Firmicutes | Mycoplasma | mobile | - | 38 | 1 | 633 | 401 |
| mmy | Firmicutes | Mycoplasma | mycoides | subsp. mycoides SC PG1 | 38 | 2 | 1017 | 469 |
| mpa | Actinobacteria | Mycobacterium | avium | paratuberculosis | 36 | 2 | 4350 | 1656 |
| mpe | Firmicutes | Mycoplasma | penetrans | - | 38 | 1 | 1037 | 472 |
| mpn | Firmicutes | Mycoplasma | pneumoniae | M129 | 38 | 3 | 689 | 356 |
| mpu | Firmicutes | Mycoplasma | pulmonis | - | 38 | 1 | 782 | 450 |
| mtc | Actinobacteria | Mycobacterium | tuberculosis | CDC1551 | 36 | 11 | 4189 | 1727 |
| mth | Euryarchaeota | Methanothermobacter | thermautotrophicus | - | 2 | 1 | 1873 | 1009 |
| mtu | Actinobacteria | Mycobacterium | tuberculosis | H37Rv | 36 | 11 | 4003 | 1867 |
| neu | Betaproteobacteria | Nitrosomonas | europaea | - | 4 | 1 | 2461 | 1405 |
| nma | Betaproteobacteria | Neisseria | meningitidis | Z2491 (serogroup A) | 18 | 14 | 1909 | 1213 |
| nme | Betaproteobacteria | Neisseria | meningitidis | MC58 (serogroup B) | 18 | 14 | 2063 | 1232 |
| oih | Firmicutes | Oceanobacillus | iheyensis | - | 1 | 1 | 3500 | 1837 |
| pab | Euryarchaeota | Pyrococcus | abyssi | - | 6 | 1 | 1781 | 1037 |
| paf | Gammaproteobacteria | Pseudomonas | aeruginosa | M18 | 28 | 6 | 5684 | 2703 |
| pai | Crenarchaeota | Pyrobaculum | aerophilum | - | 6 | 1 | 2604 | 1013 |
| pfu | Euryarchaeota | Pyrococcus | furiosus | - | 6 | 1 | 2122 | 1062 |
| pho | Euryarchaeota | Pyrococcus | horikoshii | - | 6 | 1 | 1950 | 977 |
| plu | Gammaproteobacteria | Photorhabdus | luminescens | - | 2 | 1 | 4683 | 2284 |
| pma | Cyanobacteria | Prochlorococcus | marinus | SS120 | 12 | 12 | 1883 | 985 |
| pmm | Cyanobacteria | Prochlorococcus | marinus | MED4 | 12 | 12 | 1717 | 975 |
| pmp | Gammaproteobacteria | Pasteurella | multocida | 36950 | 4 | 4 | 2098 | 1486 |
| pmt | Cyanobacteria | Prochlorococcus | marinus | MIT 9313 | 12 | 12 | 2269 | 1126 |
| poy | Firmicutes | Phytoplasma | OY | - | 4 | 1 | 749 | 335 |
| ppr | Gammaproteobacteria | Photobacterium | profundum | - | 1 | 1 | 5489 | 2671 |
| ppu | Gammaproteobacteria | Pseudomonas | putida | KT2440 | 28 | 6 | 5350 | 2699 |
| psb | Gammaproteobacteria | Pseudomonas | syringae | pv. syringae B728a | 28 | 3 | 5089 | 2655 |
| rco | Alphaproteobacteria | Rickettsia | conorii | - | 40 | 1 | 1374 | 661 |
| rps | Alphaproteobacteria | Rickettsia | prowazekii | Katsinyian | 40 | 8 | 844 | 591 |
| rpt | Alphaproteobacteria | Rhodopseudomonas | palustris | TIE-1 | 7 | 7 | 5246 | 2404 |
| rsc | Betaproteobacteria | Ralstonia | solanacearum | CFBP2957 | 8 | 4 | 5011 | 2194 |
| sag | Firmicutes | Streptococcus | agalactiae | 2603 (serotype V) | 79 | 3 | 2124 | 1137 |
| sam | Firmicutes | Staphylococcus | aureus | subsp. aureus MW2 (CA-MRSA) | 39 | 30 | 2624 | 1424 |
| san | Firmicutes | Streptococcus | agalactiae | NEM316 (serotype III) | 79 | 3 | 2094 | 1147 |
| sau | Firmicutes | Staphylococcus | aureus | subsp. aureus N315 (MRSA/VSSA) | 39 | 30 | 2614 | 1469 |
| sav | Firmicutes | Staphylococcus | aureus | subsp. aureus Mu50 (MRSA/VISA) | 39 | 30 | 2730 | 1446 |
| sco | Actinobacteria | Streptomyces | coelicolor | - | 11 | 1 | 8153 | 2457 |
| ser | Firmicutes | Staphylococcus | epidermidis | RP62A | 39 | 2 | 2525 | 1305 |
| sfl | Gammaproteobacteria | Shigella | flexneri | 301 (serotype 2a) | 9 | 4 | 4439 | 2612 |
| sfx | Gammaproteobacteria | Shigella | flexneri | 2457T (serotype 2a) | 9 | 4 | 4060 | 2541 |
| sma | Actinobacteria | Streptomyces | avermitilis | - | 11 | 1 | 7676 | 2412 |
| sme | Alphaproteobacteria | Sinorhizobium | meliloti | 1021 | 7 | 4 | 6218 | 3112 |
| smc | Firmicutes | Streptococcus | mutans | NN2025 | 79 | 2 | 1895 | 1096 |
| son | Gammaproteobacteria | Shewanella | oneidensis | - | 24 | 1 | 4467 | 2083 |
| spg | Firmicutes | Streptococcus | pyogenes | MGAS315 (serotype M3) | 79 | 16 | 1865 | 1027 |
| spm | Firmicutes | Streptococcus | pyogenes | MGAS8232 (serotype M18) | 79 | 16 | 1839 | 1024 |
| spn | Firmicutes | Streptococcus | pneumoniae | TIGR4 (virulent serotype 4) | 79 | 17 | 2105 | 1217 |
| spr | Firmicutes | Streptococcus | pneumoniae | R6 (avirulent) | 79 | 17 | 2042 | 1203 |
| sps | Firmicutes | Streptococcus | pyogenes | SSI-1 (serotype M3) | 79 | 16 | 1859 | 1021 |
| spy | Firmicutes | Streptococcus | pyogenes | SF370 (serotype M1) | 79 | 16 | 1696 | 1024 |
| stm | Gammaproteobacteria | Salmonella | enterica | subsp. enterica serovar Typhimurium LT2 | 27 | 26 | 4525 | 2940 |
| sto | Crenarchaeota | Sulfolobus | tokodaii | - | 13 | 1 | 2826 | 1050 |
| stt | Gammaproteobacteria | Salmonella | enterica | subsp. enterica serovar Typhi Ty2 | 27 | 26 | 4313 | 2755 |
| sty | Gammaproteobacteria | Salmonella | enterica | subsp. enterica serovar Typhi CT18 | 27 | 26 | 4753 | 2814 |
| sus | Acidobacteria | Candidatus Solibacter | usitatus | - | 1 | 1 | 7826 | 2001 |
| syn | Cyanobacteria | Synechocystis | sp. | PCC 6803 | 5 | 5 | 3575 | 1649 |
| syw | Cyanobacteria | Synechococcus | sp. | WH8102 | 11 | 9 | 2519 | 1210 |
| tac | Euryarchaeota | Thermoplasma | acidophilum | - | 2 | 1 | 1484 | 790 |
| tde | Spirochaetes | Treponema | denticola | - | 13 | 1 | 2767 | 1024 |
| tpa | Spirochaetes | Treponema | pallidum | subsp. pallidum Nichols | 13 | 7 | 1036 | 561 |
| tte | Firmicutes | Thermoanaerobacter | tengcongensis | - | 11 | 1 | 2588 | 1343 |
| ttl | Deinococcus-Thermus | Thermus | thermophilus | JL-18 | 6 | 4 | 2402 | 1170 |
| tvo | Euryarchaeota | Thermoplasma | volcanium | - | 2 | 1 | 1501 | 783 |
| twh | Actinobacteria | Tropheryma | whipplei | Twist | 2 | 2 | 808 | 531 |
| tws | Actinobacteria | Tropheryma | whipplei | TW08/27 | 2 | 2 | 783 | 529 |
| uur | Firmicutes | Ureaplasma | parvum | serovar 3 ATCC 700970 | 3 | 2 | 614 | 356 |
| vcr | Gammaproteobacteria | Vibrio | cholerae | O395 | 20 | 8 | 3934 | 2077 |
| vpa | Gammaproteobacteria | Vibrio | parahaemolyticus | - | 20 | 1 | 4832 | 2556 |
| vvu | Gammaproteobacteria | Vibrio | vulnificus | CMCP6 | 20 | 3 | 4433 | 2447 |
| vvy | Gammaproteobacteria | Vibrio | vulnificus | YJ016 | 20 | 3 | 5023 | 2507 |
| wbr | Gammaproteobacteria | Wigglesworthia | glossinidia | brevipalpis | 2 | 2 | 617 | 585 |
| wol | Alphaproteobacteria | Wolbachia | wMel | - | 4 | 1 | 1195 | 579 |
| wsu | Epsilonproteobacteria | Wolinella | succinogenes | - | 1 | 1 | 2043 | 1199 |
| xax | Gammaproteobacteria | Xanthomonas | axonopodis | pv. citrumelo F1 | 12 | 2 | 4181 | 1953 |
| xcb | Gammaproteobacteria | Xanthomonas | campestris | pv. campestris 8004 | 12 | 5 | 4271 | 2128 |
| xfa | Gammaproteobacteria | Xylella | fastidiosa | 9a5c | 5 | 5 | 2832 | 1281 |
| xft | Gammaproteobacteria | Xylella | fastidiosa | Temecula1 | 5 | 5 | 2036 | 1216 |
| ype | Gammaproteobacteria | Yersinia | pestis | CO92 (biovar Orientalis) | 19 | 12 | 4066 | 2627 |
| ypk | Gammaproteobacteria | Yersinia | pestis | KIM 10 (biovar Mediaevalis) | 19 | 12 | 4164 | 2484 |
| ypm | Gammaproteobacteria | Yersinia | pestis | 91001 (biovar Microtus) | 19 | 12 | 4137 | 2545 |
