# Supplementary material for: Comparative Analysis of Functional Metagenomic Annotation and the Mappability of Short Reads
Source: PLoS One. 2014 Aug 22;9(8):e105776. doi: 10.1371/journal.pone.0105776 (PMC4141809; doi:10.1371/journal.pone.0105776)
Supplement: Table S2 — Summary of all datasets generated for this study. (DOCX) [file pone.0105776.s007.docx]

**Table S2:** Summary of all datasets generated for this study.

| Index | Name | KEGG ID | Read Length (bases) | # Reads | Total Bases | Sequencing Error (%) |
| --- | --- | --- | --- | --- | --- | --- |
| 1 | Streptococcus pneumoniae ATCC 700669 | sne | 75 | 2221315 | 166598625 | 0 |
| 2 | Streptococcus pneumoniae ATCC 700669 | sne | 101 | 2221315 | 224352815 | 0 |
| 3 | Streptococcus pneumoniae ATCC 700669 | sne | 101 | 2221315 | 224352815 | 0.15 |
| 4 | Streptococcus pneumoniae ATCC 700669 | sne | 101 | 2221315 | 224352815 | 1.5 |
| 5 | Streptococcus pneumoniae ATCC 700669 | sne | 101 | 2221315 | 224352815 | 3 |
| 6 | Streptococcus pneumoniae ATCC 700669 | sne | 150 | 2221315 | 333197250 | 0 |
| 7 | Streptococcus pneumoniae ATCC 700669 | sne | 200 | 2221315 | 444263000 | 0 |
| 8 | Streptococcus pneumoniae ATCC 700669 | sne | 300 | 2221315 | 666394500 | 0 |
| 9 | Streptococcus pneumoniae ATCC 700669 | sne | 400 | 2221315 | 888526000 | 0 |
| 10 | Escherichia coli C ATCC 8739 | ecl | 101 | 4746218 | 479368018 | 0 |
| 11 | Bacteroides fragilis 638R | bfg | 101 | 5373121 | 542685221 | 0 |
| 12 | Aquifex aeolicus | aae | 101 | 1590791 | 160669891 | 0 |
| 13 | Acidobacterium capsulatum | aca | 101 | 4127356 | 416862956 | 0 |
| 14 | Bdellovibrio bacteriovorus | bba | 101 | 3782950 | 382077950 | 0 |
| 15 | Borrelia burgdorferi | bbz | 101 | 1345494 | 135894894 | 0 |
| 16 | Bifidobacterium longum | bln | 101 | 2832748 | 286107548 | 0 |
| 17 | Brucella melitensis | bme | 101 | 3294931 | 332788031 | 0 |
| 18 | Bordetella pertussis | bpe | 101 | 4086189 | 412705089 | 0 |
| 19 | Bacillus subtilis | bsu | 101 | 4215606 | 425776206 | 0 |
| 20 | Chlorobium tepidum | cte | 101 | 2154946 | 217649546 | 0 |
| 21 | Chlamydia trachomatis | ctr | 101 | 1042519 | 105294419 | 0 |
| 22 | Dehalococcoides ethenogenes | det | 101 | 1469720 | 148441720 | 0 |
| 23 | Deinococcus radiodurans | dra | 101 | 3284156 | 331699756 | 0 |
| 24 | Escherichia coli O157:H7 | ecf | 101 | 5704171 | 576121271 | 0 |
| 25 | Fusobacterium nucleatum | fnu | 101 | 2174500 | 219624500 | 0 |
| 26 | Fibrobacter succinogenes | fsu | 101 | 3842635 | 388106135 | 0 |
| 27 | Helicobacter pylori 26695 | hpy | 101 | 1667867 | 168454567 | 0 |
| 28 | Methanococcus maripaludis | mmd | 101 | 1746697 | 176416397 | 0 |
| 29 | Nanoarchaeum equitans | neq | 101 | 490885 | 49579385 | 0 |
| 30 | Porphyromonas gingivalis | pgt | 101 | 2339898 | 236329698 | 0 |
| 31 | Rhodopirellula baltica | rba | 101 | 7145576 | 721703176 | 0 |
| 32 | Sulfolobus solfataricus | sso | 101 | 2992245 | 302216745 | 0 |
| 33 | Synechococcus elongatus | syc | 101 | 2696255 | 272321755 | 0 |
| 34 | Thermotoga maritima | tma | 101 | 1860725 | 187933225 | 0 |
| 35 | Archaeoglobus fulgidus | afu | 101 | 217840 | 22001840 | 0 |
| 36 | Nostoc sp. PCC 7120 | ana | 101 | 721179 | 72839079 | 0 |
| 37 | Aeropyrum pernix | ape | 101 | 166970 | 16863970 | 0 |
| 38 | Agrobacterium tumefaciens Cereon | atu | 101 | 567426 | 57310026 | 0 |
| 39 | Agrobacterium tumefaciens WashU | atu | 101 | 567426 | 57310026 | 0 |
| 40 | Buchnera aphidicola Bp | bab | 101 | 61838 | 6245638 | 0 |
| 41 | Buchnera aphidicola Sg | bas | 101 | 64146 | 6478746 | 0 |
| 42 | Bacillus anthracis | bat | 101 | 522867 | 52809567 | 0 |
| 43 | Bordetella bronchiseptica | bbr | 101 | 533918 | 53925718 | 0 |
| 44 | Bacillus cereus ATCC 10987 | bca | 101 | 543266 | 54869866 | 0 |
| 45 | Bacillus cereus ATCC 14579 | bce | 101 | 542709 | 54813609 | 0 |
| 46 | Blochmannia floridanus | bfl | 101 | 70556 | 7126156 | 0 |
| 47 | Bacillus halodurans | bha | 101 | 420236 | 42443836 | 0 |
| 48 | Bradyrhizobium japonicum | bju | 101 | 920739 | 92994639 | 0 |
| 49 | Bordetella parapertussis | bpa | 101 | 477356 | 48212956 | 0 |
| 50 | Brucella suis | bsv | 101 | 331609 | 33492509 | 0 |
| 51 | Bacteroides thetaiotaomicron | bth | 101 | 629340 | 63563340 | 0 |
| 52 | Buchnera aphidicola APS | buc | 101 | 65573 | 6622873 | 0 |
| 53 | Clostridium acetobutylicum | cae | 101 | 414559 | 41870459 | 0 |
| 54 | Coxiella burnetii | cbu | 101 | 203268 | 20530068 | 0 |
| 55 | Chlamydophila caviae | cca | 101 | 118136 | 11931736 | 0 |
| 56 | Caulobacter crescentus | ccr | 101 | 401695 | 40571195 | 0 |
| 57 | Corynebacterium diphtheriae | cdw | 101 | 253069 | 25559969 | 0 |
| 58 | Corynebacterium efficiens | cef | 101 | 321951 | 32517051 | 0 |
| 59 | Corynebacterium glutamicum 13032 | cgb | 101 | 328271 | 33155371 | 0 |
| 60 | Corynebacterium glutamicum | cgt | 101 | 336330 | 33969330 | 0 |
| 61 | Campylobacter jejuni | cju | 101 | 162812 | 16444012 | 0 |
| 62 | Chlamydia muridarum | cmu | 101 | 108046 | 10912646 | 0 |
| 63 | Chlamydia pneumoniae AR39 | cpa | 101 | 122986 | 12421586 | 0 |
| 64 | Clostridium perfringens | cpe | 101 | 308574 | 31165974 | 0 |
| 65 | Chlamydia pneumoniae J138 | cpj | 101 | 122657 | 12388357 | 0 |
| 66 | Chlamydia pneumoniae CWL029 | cpn | 101 | 123023 | 12425323 | 0 |
| 67 | Chlamydophila pneumoniae TW183 | cpt | 101 | 122594 | 12381994 | 0 |
| 68 | Clostridium tetani | ctc | 101 | 287334 | 29020734 | 0 |
| 69 | Chromobacterium violaceum | cvi | 101 | 475108 | 47985908 | 0 |
| 70 | Desulfovibrio vulgaris | dvl | 101 | 366140 | 36980140 | 0 |
| 71 | Escherichia coli O6 | ecc | 101 | 523143 | 52837443 | 0 |
| 72 | Escherichia coli EDL933 | ece | 101 | 562053 | 56767353 | 0 |
| 73 | Escherichia coli K12 | eco | 101 | 463968 | 46860768 | 0 |
| 74 | Enterococcus faecalis | efa | 101 | 335998 | 33935798 | 0 |
| 75 | Geobacter sulfurreducens | gsu | 101 | 381414 | 38522814 | 0 |
| 76 | Gloeobacter violaceus | gvi | 101 | 465902 | 47056102 | 0 |
| 77 | Halobacterium sp. NRC-1 | hal | 101 | 257101 | 25967201 | 0 |
| 78 | Haemophilus ducreyi | hdu | 101 | 169896 | 17159496 | 0 |
| 79 | Helicobacter hepaticus | hhe | 101 | 179915 | 18171415 | 0 |
| 80 | Haemophilus influenzae | hiz | 101 | 193231 | 19516331 | 0 |
| 81 | Helicobacter pylori J99 | hpj | 101 | 164384 | 16602784 | 0 |
| 82 | Leptospira interrogans L1-130 | lic | 101 | 462737 | 46736437 | 0 |
| 83 | Leptospira interrogans 56601 | lil | 101 | 469814 | 47451214 | 0 |
| 84 | Listeria innocua | lin | 101 | 309312 | 31240512 | 0 |
| 85 | Lactobacillus johnsonii | ljo | 101 | 199268 | 20126068 | 0 |
| 86 | Lactococcus lactis | llm | 101 | 252948 | 25547748 | 0 |
| 87 | Listeria monocytogenes F2365 | lmf | 101 | 290519 | 29342419 | 0 |
| 88 | Listeria monocytogenes EGD | lmo | 101 | 294453 | 29739753 | 0 |
| 89 | Lactobacillus plantarum | lpj | 101 | 319776 | 32297376 | 0 |
| 90 | Methanosarcina acetivorans | mac | 101 | 575150 | 58090150 | 0 |
| 91 | Mycobacterium bovis | mbm | 101 | 435039 | 43938939 | 0 |
| 92 | Mycoplasma gallisepticum | mga | 101 | 101280 | 10229280 | 0 |
| 93 | Mycoplasma genitalium | mge | 101 | 58008 | 5858808 | 0 |
| 94 | Methanococcus jannaschii | mja | 101 | 173993 | 17573293 | 0 |
| 95 | Methanopyrus kandleri | mka | 101 | 169497 | 17119197 | 0 |
| 96 | Mycobacterium leprae | mlb | 101 | 326808 | 33007608 | 0 |
| 97 | Rhizobium loti | mlo | 101 | 759630 | 76722630 | 0 |
| 98 | Methanosarcina mazei | mma | 101 | 409635 | 41373135 | 0 |
| 99 | Mycoplasma mobile | mmo | 101 | 77708 | 7848508 | 0 |
| 100 | Mycoplasma mycoides | mmy | 101 | 121171 | 12238271 | 0 |
| 101 | Mycobacterium paratuberculosis | mpa | 101 | 482979 | 48780879 | 0 |
| 102 | Mycoplasma penetrans | mpe | 101 | 135864 | 13722264 | 0 |
| 103 | Mycoplasma pneumoniae | mpn | 101 | 81640 | 8245640 | 0 |
| 104 | Mycoplasma pulmonis | mpu | 101 | 96388 | 9735188 | 0 |
| 105 | Mycobacterium tuberculosis CDC1551 | mtc | 101 | 440384 | 44478784 | 0 |
| 106 | Methanobacterium thermautotrophicum | mth | 101 | 175138 | 17688938 | 0 |
| 107 | Mycobacterium tuberculosis H37Rv | mtu | 101 | 441154 | 44556554 | 0 |
| 108 | Nitrosomonas europaea | neu | 101 | 281210 | 28402210 | 0 |
| 109 | Neisseria meningitidis A | nma | 101 | 218441 | 22062541 | 0 |
| 110 | Neisseria meningitidis B | nme | 101 | 227236 | 22950836 | 0 |
| 111 | Oceanobacillus iheyensis | oih | 101 | 363053 | 36668353 | 0 |
| 112 | Pyrococcus abyssi | pab | 101 | 176857 | 17862557 | 0 |
| 113 | Pseudomonas aeruginosa | paf | 101 | 632776 | 63910376 | 0 |
| 114 | Pyrobaculum aerophilum | pai | 101 | 222243 | 22446543 | 0 |
| 115 | Pyrococcus furiosus | pfu | 101 | 190826 | 19273426 | 0 |
| 116 | Pyrococcus horikoshii | pho | 101 | 173851 | 17558951 | 0 |
| 117 | Photorhabdus luminescens | plu | 101 | 568899 | 57458799 | 0 |
| 118 | Prochlorococcus marinus SS120 | pma | 101 | 175108 | 17685908 | 0 |
| 119 | Prochlorococcus marinus CCMP1378 | pmm | 101 | 165799 | 16745699 | 0 |
| 120 | Pasteurella multocida | pmp | 101 | 234952 | 23730152 | 0 |
| 121 | Prochlorococcus marinus MIT9313 | pmt | 101 | 241088 | 24349888 | 0 |
| 122 | Phytoplasma Onion yellows | poy | 101 | 85310 | 8616310 | 0 |
| 123 | Photobacterium profundum | ppr | 101 | 640328 | 64673128 | 0 |
| 124 | Pseudomonas putida | ppu | 101 | 618187 | 62436887 | 0 |
| 125 | Pseudomonas syringae | psb | 101 | 609370 | 61546370 | 0 |
| 126 | Rickettsia conorii | rco | 101 | 126876 | 12814476 | 0 |
| 127 | Rickettsia prowazekii | rps | 101 | 111146 | 11225746 | 0 |
| 128 | Rhodopseudomonas palustris | rpt | 101 | 574405 | 58014905 | 0 |
| 129 | Ralstonia solanacearum | rsc | 101 | 558077 | 56365777 | 0 |
| 130 | Streptococcus agalactiae V | sag | 101 | 216027 | 21818727 | 0 |
| 131 | Staphylococcus aureus MW2 | sam | 101 | 282047 | 28486747 | 0 |
| 132 | Streptococcus agalactiae III | san | 101 | 221149 | 22336049 | 0 |
| 133 | Staphylococcus aureus N315 | sau | 101 | 283947 | 28678647 | 0 |
| 134 | Staphylococcus aureus Mu50 | sav | 101 | 290364 | 29326764 | 0 |
| 135 | Streptomyces coelicolor | sco | 101 | 905485 | 91453985 | 0 |
| 136 | Staphylococcus epidermidis | ser | 101 | 264384 | 26702784 | 0 |
| 137 | Shigella flexneri 2a 301 | sfl | 101 | 482882 | 48771082 | 0 |
| 138 | Shigella flexneri 2a 2457T | sfx | 101 | 459936 | 46453536 | 0 |
| 139 | Streptomyces avermitilis | sma | 101 | 911990 | 92110990 | 0 |
| 140 | Rhizobium meliloti | sme | 101 | 669170 | 67586170 | 0 |
| 141 | Streptococcus mutans | smc | 101 | 201359 | 20337259 | 0 |
| 142 | Shewanella oneidensis | son | 101 | 513142 | 51827342 | 0 |
| 143 | Streptococcus pyogenes MGAS315 | spg | 101 | 190053 | 19195353 | 0 |
| 144 | Streptococcus pyogenes MGAS8232 | spm | 101 | 189502 | 19139702 | 0 |
| 145 | Streptococcus pneumoniae TIGR4 | spn | 101 | 216085 | 21824585 | 0 |
| 146 | Streptococcus pneumoniae R6 | spr | 101 | 203862 | 20590062 | 0 |
| 147 | Streptococcus pyogenes SSI-1 | sps | 101 | 189428 | 19132228 | 0 |
| 148 | Streptococcus pyogenes M1 | spy | 101 | 185245 | 18709745 | 0 |
| 149 | Salmonella typhimurium | stm | 101 | 495138 | 50008938 | 0 |
| 150 | Sulfolobus tokodaii | sto | 101 | 269476 | 27217076 | 0 |
| 151 | Salmonella enterica | stt | 101 | 479197 | 48398897 | 0 |
| 152 | Salmonella typhi | sty | 101 | 513372 | 51850572 | 0 |
| 153 | Solibacter usitatus | sus | 101 | 996564 | 100652964 | 0 |
| 154 | Synechocystis sp. PCC6803 | syn | 101 | 394702 | 39864902 | 0 |
| 155 | Synechococcus sp. WH8102 | syw | 101 | 243443 | 24587743 | 0 |
| 156 | Thermoplasma acidophilum | tac | 101 | 156491 | 15805591 | 0 |
| 157 | Treponema denticola | tde | 101 | 284321 | 28716421 | 0 |
| 158 | Treponema pallidum | tpa | 101 | 113802 | 11494002 | 0 |
| 159 | Thermoanaerobacter tengcongensis | tte | 101 | 268945 | 27163445 | 0 |
| 160 | Thermus thermophilus | ttl | 101 | 231122 | 23343322 | 0 |
| 161 | Thermoplasma volcanium | tvo | 101 | 158481 | 16006581 | 0 |
| 162 | Tropheryma whipplei Twist | twh | 101 | 92731 | 9365831 | 0 |
| 163 | Tropheryma whipplei TW08/27 | tws | 101 | 92594 | 9351994 | 0 |
| 164 | Ureaplasma parvum | uur | 101 | 75172 | 7592372 | 0 |
| 165 | Vibrio cholerae | vcr | 101 | 413530 | 41766530 | 0 |
| 166 | Vibrio parahaemolyticus | vpa | 101 | 516577 | 52174277 | 0 |
| 167 | Vibrio vulnificus CMCP6 | vvu | 101 | 512670 | 51779670 | 0 |
| 168 | Vibrio vulnificus YJ016 | vvy | 101 | 526009 | 53126909 | 0 |
| 169 | Wigglesworthia brevipalpis | wbr | 101 | 70301 | 7100401 | 0 |
| 170 | Wolbachia sp. wMel | wol | 101 | 126779 | 12804679 | 0 |
| 171 | Wolinella succinogenes | wsu | 101 | 211036 | 21314636 | 0 |
| 172 | Xanthomonas axonopodis | xax | 101 | 496747 | 50171447 | 0 |
| 173 | Xanthomonas campestris | xcb | 101 | 514871 | 52001971 | 0 |
| 174 | Xylella fastidiosa 9a5c | xfa | 101 | 273175 | 27590675 | 0 |
| 175 | Xylella fastidiosa 700964 | xft | 101 | 252115 | 25463615 | 0 |
| 176 | Yersinia pestis CO92 | ype | 101 | 482986 | 48781586 | 0 |
| 177 | Yersinia pestis KIM | ypk | 101 | 470175 | 47487675 | 0 |
| 178 | Yersinia pestis Medievalis | ypm | 101 | 480322 | 48512522 | 0 |
| Total |  |  |  | 143163496 | 15.8367284 GB |  |
