# Supplementary material for: Comparative Analysis of Functional Metagenomic Annotation and the Mappability of Short Reads
Source: PLoS One. 2014 Aug 22;9(8):e105776. doi: 10.1371/journal.pone.0105776 (PMC4141809; doi:10.1371/journal.pone.0105776)
Supplement: Text S1 — An analysis of E-value cutoff schemes and the effect of low-complexity filtering on typical E-value distributions, and an analysis of reads originating from multiple genomic regions. (DOCX) [file pone.0105776.s013.docx]

**Comparative analysis of functional metagenomic annotation and the mappability of short reads**

**Rogan Carr^1^ and Elhanan Borenstein^1,2,3,*^**

^1^ Department of Genome Sciences, University of Washington, Seattle, WA, USA
^2^ Department of Computer Science and Engineering, University of Washington, Seattle, WA, USA
^3^ Santa Fe Institute, Santa Fe, NM, USA
^*^ Corresponding author: elbo@uw.edu

**Supporting Information**

**Assessing E-value cutoff schemes and examining the effect of low-complexity filters**

To remove spurious matches that were identified by chance from a set of alignments identified by a translated BLAST search for a set of reads, it is common to use a maximal E-value cutoff to filter out low-scoring alignments. The choice of this cutoff value represents a tradeoff between recall and precision, with higher cutoff values resulting in higher recall but also in reduced precision (and vice versa). However, since the calculated E-value for each alignment depends also on the length of the read, read length should be considered when the E-value cutoff is determined. Indeed, examining the distribution of E-values obtained for the 101-bp read dataset derived from *S. pneumoniae*, it is clear that correct matches may have E-values close to 1. In contrast, the distribution of E-values obtained for the corresponding 400-bp dataset demonstrates that correct matches to such long reads typically have E-values below 10^-20^ (Figure S2). These distributions show that higher precisions can be achieved at little cost to recall by using read length-specific E-value cutoffs (see also Figure S1).

Figure S2b further demonstrates that the E-value distribution from the translated BLAST search is bimodal. This could be the outcome of filtering low-complexity sequences. Translated BLAST with default parameters includes a low-complexity filter (SEG [1]) that masks regions of the query sequence determined to be of low compositional complexity. In the case of short reads, this mask can affect a significant portion of the read, resulting in a reduced score (and therefore a higher E-value) for matches to the correct protein, or a higher score for matches to an incorrect protein. To examine the effect of this low-complexity filter on the annotation of short sequencing reads, we reran the BLAST searches for the 6 different read length datasets derived from *S. pneumonia* (Table S2) with the SEG filter turned off. We found that without low-complexity filtering, the distribution of E-values for the 400-bp reads is no longer multimodal. Moreover, the annotation accuracies obtained without the low complexity filter increased slightly. For example, for the 101-bp simulation with the strain present in the database and the *top gene* protocol, higher recall (97% vs. 94%) and a similar precision (97%) to those obtained with the SEG filter were found. Similar results were obtained regardless of read length or phylogenetic coverage.

**Mappability of partially overlapping reads**

To determine the minimal number of gene-coding bases necessary to correctly map short sequencing reads to their gene and KO of origin, we examined simulated sequencing reads that overlap both a KO gene and an intergenic region. By averaging over all 101-bp datasets used in this study and analyzed with the *top gene* protocol, we identified a marked transition for correctly identifying a KO gene at 51 bases (Figure S5). Furthermore, once 55 bases of the read overlap the gene, the ability to correctly identify the KO is mostly recovered and additional overlapping bases add only small gains in recall. We observed similar results regardless of the database phylogenetic coverage. Notably, however, this analysis is based on using an E-value cutoff of 1, and is therefore dependent on database size; the use of a minimal score rather than an E-value cutoff might increase the ability to identify KO genes with shorter overlaps.

# Supporting References

1. Wootton JC, Federhen S (1996) Analysis of compositionally biased regions in sequence databases. Methods Enzymol 266: 554–571.
